# Mapping targets for small nucleolar RNAs in yeast

**DOI:** 10.12688/wellcomeopenres.14735.2

**Published:** 2018-11-22

**Authors:** Tatiana Dudnakova, Hywel Dunn-Davies, Rosie Peters, David Tollervey

**Affiliations:** 1 Wellcome Centre for Cell Biology, University of Edinburgh, Edinburgh, EH9 3BF, UK

**Keywords:** small nucleolar RNA, snoRNA, RNA-RNA interaction, RNA-protein interaction, UV cross-linking

## Abstract

**Background:** Recent analyses implicate changes in the expression of the box C/D class of small nucleolar RNAs (snoRNAs) in several human diseases.

**Methods:** Here we report the identification of potential novel RNA targets for box C/D snoRNAs in budding yeast, using the approach of UV crosslinking and sequencing of hybrids (CLASH) with the snoRNP proteins Nop1, Nop56 and Nop58. We also developed a bioinformatics approach to filter snoRNA-target interactions for bona fide methylation guide interactions.

**Results:** We recovered 241,420 hybrids, out of which 190,597 were classed as reproducible, high energy hybrids. As expected, the majority of snoRNA interactions were with the ribosomal RNAs (rRNAs). Following filtering, 117,047 reproducible hybrids included 51 of the 55 reported rRNA methylation sites. The majority of interactions at methylation sites were predicted to guide methylation. However, competing, potentially regulatory, binding was also identified. In marked contrast, following CLASH performed with the RNA helicase Mtr4 only 7% of snoRNA-rRNA interactions recovered were predicted to guide methylation. We propose that Mtr4 functions in dissociating inappropriate snoRNA-target interactions. Numerous snoRNA-snoRNA interactions were recovered, indicating potential cross regulation. The snoRNAs snR4 and snR45 were recently implicated in site-directed rRNA acetylation, and hybrids were identified adjacent to the acetylation sites. We also identified 1,368 reproducible snoRNA-mRNA interactions, representing 448 sites of interaction involving 39 snoRNAs and 382 mRNAs. Depletion of the snoRNAs U3, U14 or snR4 each altered the levels of numerous mRNAs. Targets identified by CLASH were over-represented among these species, but causality has yet to be established.

**Conclusions:** Systematic mapping of snoRNA-target binding provides a catalogue of high-confidence binding sites and indicates numerous potential regulatory interactions.

## Introduction

The small nucleolar RNAs (snoRNAs) are an abundant class of stable RNAs, most of which act as guides for site-specific RNA modification. Most members of the box C/D class of snoRNAs select sites of ribose 2’- *O*-methylation via extended regions of perfect complementarity with target sites (≥12 bp), in which the nucleotide to be modified is placed exactly 5 bp from the conserved box D or box D’ motifs within the snoRNA (reviewed in ( Tollervey & Kiss, 1997; Watkins & Bohnsack, 2012)). The box C/D snoRNAs associate with a group of four common proteins, Nop56, Nop58, Snu13 and the methyl-transferase Nop1 (Fibrillarin in humans). The snoRNAs have a partially symmetrical structure, in which stem structures bring together the highly conserved, terminal box C (RUGAUGA, R = A or G) and box D (CUGA) sequences and the related but less conserved, internal box C’ and box D’ elements. These stem structures include a K-turn structural motif that is bound by the small protein Snu13. *In vitro* structural analysis indicated that the box C/D stem is also bound by Nop58, while the box C’/D’ stem is bound by the homologous Nop56 protein. Each region is bound by a copy of Nop1, so the regions flanking either box D, box D’ or both can function as methylation guides. However, guide function has a strict requirement for a long region of perfect between the snoRNA and the target RNA, which extends to box D or D’. This implies that strong snoRNA base pairing could occur without eliciting target RNA methylation. Indeed, a small number of box C/D snoRNAs have essential functions in ribosome synthesis that require snoRNA/pre-rRNA base pairing without associated RNA methylation. These snoRNAs include U3/snR17 and U14/snR128 in yeast and U3, U14 and U8 in vertebrates (reviewed by Watkins & Bohnsack (2012)). In yeast, all known sites of snoRNA-directed methylation are in the 18S and 25S rRNAs, whereas human snoRNAs can additionally direct methylation of other small RNAs, including spliceosomal small nuclear RNAs (snRNAs) and other snoRNAs.

In yeast, the complete set of rRNA modifications have likely been identified ( Taoka *et al.*, 2016; Yang *et al.*, 2016). Bioinformatics approaches have been used to predict snoRNA binding sites in several systems, particularly where this is associated with methylation ( Jorjani *et al*., 2016; Lowe & Eddy, 1999; Lu *et al*., 2016; Omer *et al*., 2000). For a listing of yeast snoRNA-target interactions see ( https://www-snorna.biotoul.fr/) ( Lestrade & Weber, 2006). In addition, a number of recent reports have described methods for the identification of RNA-RNA interactions through proximity ligation followed by sequencing of the products of reverse transcription and PCR amplification (RT-PCR) ( Gumienny *et al.*, 2017; Kudla *et al.*, 2011; Sharma *et al.*, 2016; Sugimoto *et al.*, 2015). In the crosslinking and sequencing of hybrids (CLASH) approach, stringent tandem affinity purification including denaturing conditions is used to recover only covalent RNA-protein interactions ( Kudla *et al*., 2011). Here we report the application of CLASH to the identification of novel snoRNA-target interactions in yeast cells.

## Methods

### Yeast culture and manipulation

All yeast analyses were performed in strains derived from (BY4741, *MAT*a; *his3Δ1*; *leu2Δ0*; *met15Δ0*; *ura3Δ0*). Growth, handling, and transformation of yeast involved standard techniques. HTP-tagged Nop1, Nop56, Nop58 and Mtr4 strains constructed in our lab in the background of BY4741 were previously described ( Granneman *et al.*, 2009; Delan-Forino *et al.*, 2017). Oligonucleotides are listed in Supplementary Table 1.

Initial steps of yeast culture growing and UV crosslinking for CLASH are as previously described for CRAC ( Tuck & Tollervey, 2013). In brief, yeast cultures were grown to OD _600_=0.5 and crosslinked (254 nm, 100 s). For RNA sequencing three transformants of *P _GAL_::SNR17A snr17B∆* U3mut, snr4∆, snr45∆ and snr78-78∆ were grown in YNB supplemented with Formedium CSM (complete or –Trp) and 2% w/v glucose. BY4741 was grown as a control. Cultures were grown from OD600 ~0.1 until they reached OD600 ~0.5, at which point all samples were pelleted by centrifugation at 4°C at 1940xg for 3min. “Standard” samples were resuspended in 1ml 1X PBS, then centrifuged and the pellets frozen. “Ice” samples were resuspended in 10ml 4°C 1X PBS and incubated for 20min on ice before being pelleted and frozen.

### Construction of strains expressing mutant U3, strains with SNR4 and SNR45 depletion

U3 is encoded in *S. cerevisiae* by 2 redundant genes *SNR17A* and *SNR17B*. To assess U3 functions we deleted the *SNR17B* genetic locus and placed the expression of *SNR17A* under the control of a repressible *P _GAL_* promoter. To create *kanMX6-P _GAL1_-SNR17A snr17B∆* strains, genetic manipulations were carried out in a standard manner as described ( Longtine *et al.*, 1998) using corresponding pFA6a–MX6 plasmids. The *snr4::KanMX6* strains were created using similar methods.

Double snR4 snR45 depletion mutants were created with one snoRNA gene deleted and the other placed under *P _GAL1_* transcriptional control. Primers oRP-063 –oRP066 (Supplementary Methods_d1) were used to PCR amplify pFA6a-His3MX6-PGAL1, and this was transformed into the reciprocal snoRNA deletion strain as described above, to create strains *HIS3MX6-P _GAL1_-SNR4 snr45::KanMX6*, and *HISMX6-P _GAL1_-SNR45 snr4::KanMX6*.

Primers used for snoRNA depletion:

SNR17B_dFP

GTAAAGAGGTAAGGATGTTAATATTGCCGTGGAAAAAATTGCAACGAGAGCGGATCCCCGGGTTAATTAA

SNR17B_dRP

ATTAAAATACTAAGTATAATGCGGCTCCAAAATACTGAATCAAACCTTTGGAATTCGAGCTCGTTTAAAC

SNR4_delFP

TAGTTTTTTTGTCATTGATCTTTTCATTTTTTTATTTCAAAATCCCCATCGGATCCCCGGGTTAATTAAG

SNR4_delRP

ACCCAGGTGAGACTGGATGCTCCATAGATTCCAAGATTTACGTAAGAATTGAATTCGAGCTCGTTTAAAC

To study U3 binding domains we generated a plasmid from which the truncated mutant U3 with deleted helixes 2 and 4 and helix 3 replaced with snR77 mRNA binding sequence was expressed under the endogenous promoter. Boxes B, C, C’ and D were unaltered. The sequence containing the endogenous *P _SNR17A_* promoter and mutant U3 flanked by XbaI and EcoRI restriction sites was cloned into pRS413 (centromeric, -His) plasmid ( Frazer & O’Keefe, 2007) to obtain U3 mutant construct (U3mut). For complementation experiments we cloned the endogenous PSNR17A promoter and wild type SNR17A into the same vector (U3wt).

Sequence used to create U3mut expressing vector:

TATTTCTTTCTAGAGTTTCAAAAAAAATATTGATTCTTTTTTTATAAAAATATCAGTAGTATGTATGGGCTGATTGTATGGTTTATACAGGCCGTCAAAATTTTTTCACCCCCCCATACCCCACATACCTTTTACTATTAACCCTGATTTTTTTTCTTTTCACATACAGCGCCTTAAGGCGAAGGCAAATCCTGAAAATTTTCTCATTTGCTTTCCCCCACCAGACATATATAAAGGCTTTGTATTCTGCTGTCAATTAGATTTAGTACATCTTTTCTCTTATGTTTTCTTCTTGTTTCTACTTAAAATCTGTGTCGACGTACTTCATAGGATCATTTCTATAGGAATCGTCACTCTTTGACTCTTCAAAAGAGCCACTGAATCCAACTTGGTTGATGAGTCCCATAACCTTTGTACCCCAGAGTGAGAAACCGGCGCGATGATCTTGAATATGATGATTATAACAAAAACAAGTTTTTGCTCTAGTGGGTACAAATGGCAGTCTGACAAGTTAACCACTTTTTTCCTTTTCTAAATTGTTTAAAACCAAAGGTTTGGTTTTCAGTTAAGAAATTGGATTAGTTGGTGTGTAAGTATAATTAAATGTAGTGAATTCATCATTTA

Three *kanMX6-P _GAL1_::SNR17A snr17B∆* clones containing pRS413(His)_U3mut plasmid were selected on –His SD medium. Cultures were grown in –His SD 2% Galactose overnight, then transferred to –His SD 2% Glucose, diluted to OD _600_=0.1 and grown for 36 h to deplete the chromosomally expressed snoRNAs. Strains *HIS3MX6-P _GAL1_-SNR4 snr45::KanMX6*, and *HISMX6-P _GAL1_-SNR45 snr4::KanMX6* were depleted of corresponding snoRNAs in similar manner.

### RNA isolation

Cells were harvested and total RNA was isolated using hot Phenol-GTC lysis. Briefly: cells were disrupted by vortexing with zirconium beads and proteins were denatured in GTC:Phenol pH4 (1:1) mixture for 5 min at 65°C. Chloroform:IAA (24:1) was added and, after centrifugation, the aqueous phase was collected. RNA was precipitated, resuspended in RNase free water and stored at -70°C. The quality and quantity of RNA preparation was assessed using an Agilent 2100 Bioanalyzer System with RNA Nano Chips and RNA 6000 Nano Reagents (Agilent Technologies).

### Northern blotting for pre-rRNA analyses

A total of 5 μg or 10 μg of samples were combined with the recommended volume of glyoxal as per the protocol for the Ambion Northern Max-Gly Kit. Samples were electrophoresed on a 1.2% w/v agarose 1x BPTE (10 mM PIPES; 30 mM Bis-Tris; 1 mM EDTA) gel at 50 V overnight at 4°C in 1x BPTE buffer. The gel was treated for 20 min with 75 μM NaOH, followed by 20 min in 0.5 M Tris pH 7.5 plus 1.5 M NaCl. It was then washed with 6X SSC (0.5 M NaCl and 50 mM Na _3_C _6_H _5_O _7_, pH 7) for 20min. RNA was transferred onto a GE Healthcare Hybond-N+ membrane overnight at room temperature by capillary transfer. RNA was immobilized on the membrane by UV cross-linking at 120 mJ cm ^-2^. Results of northern blotting are shown on figshare ( Dudnakova *et al*., 2018).

The probes were labelled with [γ ^32^P]-ATP using T4 PNK (NEB) and hybridized overnight in 20X SSC (3M NaCl and 0.3 M sodium citrate, pH7) with Denhardt hybridization buffer (100x solution: 2% w/v Ficoll 400, 300 mM NaCl, 2% w/v Polyvinylpyrrolidone, 2% w/v BSA). The membrane was then exposed to a phosphor screen, the signal was detected using a fluorescent imaging analyzer (FLA-5000 scanner, Fujifilm).

### RNASeq cDNA Library preparation

From total RNA samples (10 µg total RNA), poly(A)-tailed RNAs were selected using Dynabeads™ mRNA Purification Kit (Thermo Fisher Scientific) according to manufacturer’s protocol. RNA-seq cDNA libraries were prepared with NEBNext® Ultra™ Directional RNA Library Prep Kit for Illumina according to the instruction manual provided ( https://international.neb.com/-/media/catalog/datacards-or-manuals/manuale7420.pdf) using 50-100 ng Poly(A)-selected RNA samples as starting material. NEBNext Multiplex Oligos (NEB) adaptors and primers were used to multiplex cDNA Illumina libraries.

### Quantitative PCR

Total RNA was DNase-treated as per the Ambion Turbo DNase protocol. RT was carried out from 0.5μg of treated RNA with a RETROscripts Reverse Transcriptase kit and Random decamer primers (Ambion). The RT sample was then diluted to either 1 ng μl ^-1^ or 0.1 ng μl ^-1^ and 4 μl was used with Takara Bio SYBR Premier Ex Taq 2x mix and the corresponding set of primers (Supplementary Document 3). This was then amplified by qPCR by the Agilent Stratagene Mx3005P, using the following method for SYBR Green with dissociation curve: 95°C for 1min, followed by 40 cycles (95°C for 15 s, 55°C for 15 s, and 72°C for 15 s), followed by 95°C for 1 min, 55°C for 30 s and 95°C for 30 s.

### Crosslinking, preprocessing and aligning of Illumina sequence data

CLASH experiments using yeast cells were performed on cultures grown in synthetic dextrose (SD) medium with 2% glucose, lacking Trp to OD600 0.5, or following to synthetic medium containing 2% v/v ethanol plus 2% v/v glycerol for 20 min. Actively growing cells were cross-linked in culture medium ( Granneman *et al.*, 2011) and processed for CLASH as previously described ( Granneman *et al.*, 2009; Helwak *et al.*, 2013; Kudla *et al.*, 2011). Briefly, cells were lysed in buffer “A” containing 50 mM Tris-HCl pH 7.8, 2 mM MgCl _2_, 150 mM NaCl, 0.4% NP-40 (all chemicals acquired from Sigma-Aldrich) with RNAsin Ribonuclease Inhibitor (Promega). RNA-protein complexes were isolated by binding to an IgG column (GE), washed in buffer A and released by TEV (Promega) cleavage. RNAs were partially digested using RNaceIT Ribonuclease Cocktail (Agilent). RNA-protein complexes were bound in denaturing conditions (6M Guanidinium HCl in buffer A) to a nickel affinity column (Ni-NTA agarose, QIAGEN). RNA end processing, radiolabeling and linker ligation were performed on the nickel column. 3’ linker ligation and simultaneous internal hybrid ligation (ssRNA Ligase I, NEB) was carried out without ATP in ligation buffer. Subsequently, complexes were eluted (200mM Imidazole, 100mM DTT) and resolved on NuPage 4–12% gradient gels (Invitrogen, Thermo Fisher Scientific) transferred to nitrocellulose (GE), identified by autoradiography and excised. The proteins were then digested with proteinase K (Roche) and the cDNA library was created and amplified from the acquired RNAs using RT-PCR (Superscript III RT, Invitrogen; LA Taq, TaKara). 5’ linkers used to prepare libraries contain a barcode, enabling samples to be multiplexed for sequencing, and a random 3 nt sequence in order to remove PCR duplicates by collapsing identical sequences during data analysis.

3’ linker

miRCat-33 linker (IDT) AppTGGAATTCTCGGGTGCCAAG/ddC/

5’ linkers (barcode marked in bold, random nucleotides as N)

L5Aa invddT-ACACrGrArCrGrCrUrCrUrUrCrCrGrArUrCrUrNrNrNr **UrArArGrC**-OH

L5Ab invddT-ACACrGrArCrGrCrUrCrUrUrCrCrGrArUrCrUrNrNrNr **ArUrUrArGrC**-OH

L5Ac invddT-ACACrGrArCrGrCrUrCrUrUrCrCrGrArUrCrUrNrNrNr **GrCrGrCrArGrC**-OH

L5Bb invddT-ACACrGrArCrGrCrUrCrUrUrCrCrGrArUrCrUrNrNrNr **GrUrGrArGrC**-OH

L5Bc invddT-ACACrGrArCrGrCrUrCrUrUrCrCrGrArUrCrUrNrNrNr **CrArCrUrArGrC**-OH

L5Bd invddT-ACACrGrArCrGrCrUrCrUrUrCrCrGrArUrCrUrNrNrNr **UrCrUrCrUrArGrC**-OH

L5Ca invddT-ACACrGrArCrGrCrUrCrUrUrCrCrGrArUrCrUrNrNrNr **CrUrArGrC**- OH

L5Cb invddT-ACACrGrArCrGrCrUrCrUrUrCrCrGrArUrCrUrNrNrNr **GrGrArGrC**-OH

L5Cc invddT-ACACrGrArCrGrCrUrCrUrUrCrCrGrArUrCrUrNrNrNr **ArCrTrCrArGrC**-OH

L5Cd invddT-ACACrGrArCrGrCrUrCrUrUrCrCrGrArUrCrUrNrNrNr **GrArCrTrTrArGrC**-OH

PCR primers

miRCat-33 primer (IDT) CCTTGGCACCCGAGAATT primer for RT

PE_miRCat_PCR CAAGCAGAAGACGGCATACGAGATCGGTCTCGGCATTCCTGGCCTTGGCACCCGAGAATTCC library amplification

P5 AATGATACGGCGACCACCGAGATCTACACTCTTTCCCTACACGACGCTCTTCCGATCT library amplification

### RNA-IP for U3 mutations

Strains expressing chromosomally encoded HTP tagged Nop1 or Rrp9 and mutant U3 (expressed from the plasmid) were grown to OD _600_ 0.5, Cells were collected, lysed in buffer A and RNA protein complexes purified on the IgG column (materials as for CLASH, see above). RNA was extracted using Phenol/Chloroform and analyzed by Northern Blot using anti-U3 DNA oligo probe 5 ’ –CTATAGAAATGATCC, as described previously ( Dandekar & Tollervey, 1989).

For hybridization, 15 ml of 20X SSC (3 M NaCl and 0.3 M sodium citrate, pH7) was mixed with 2.5 ml 100X Denhardt hybridization buffer (2% w/v Ficoll 400, 300 mM NaCl, 2% w/v Polyvinylpyrrolidone, 2% w/v BSA) and 1.25 ml 20% w/v SDS. This was incubated at 50°C and filter-sterilized. This solution was added to the membrane in a plastic box, and incubated for 1h at 37°C with shaking. To make hybridization probes, 1μl (10U) of T4 polynucleotide kinase (PNK) was mixed with 1 μl oligo (at 10 μM), 1.5 μl 10X PNK buffer, 9 μl H _2_O and 2.5 μl γ-ATP ( ^32^P). This was incubated at 37°C for 40 min, then purified on a Roche Mini Quick Spin Oligo column by centrifugation at 1000 g for 1 min. The labelled probe was added to fresh hybridization buffer and incubated with the membrane overnight at 37°C. The membrane was subsequently washed with 6X SSC plus 0.1% w/v SDS at 37°C, a total of three times for 10 min each. The membrane was then exposed to a phosphor screen and the signal detected using a fluorescent imaging analyzer (FLA-5000 scanner, Fujifilm).

### PolyA selection

To select for poly(A) tailed RNAs, the NEBNext poly(A) mRNA Magnetic Isolation Module kit was used, and the protocol followed. A total of 20 μl Oligo d(T) _25_ beads per sample were washed twice with 100 μl 2x RNA Binding Buffer. RNA samples were DNase-treated with Promega 10X RQ1 buffer, 1 unit RQ1 DNase and 1 unit of Promega RNasin and incubated at room temperature for 30 min. The reaction was stopped by adding 50 mM EDTA and incubating on ice, followed by addition of 10 mM Tris pH7.8 and 100 mM NaOAc, and transfer into RNA Phenol:Chloroform:IAA (25:24:1), pH4. The RNA isolation protocol was followed from this step. A total of 2 μg total RNA, as measured by Agilent Bioanalyzer RNA chip was diluted to a total of 50 μl in nuclease-free water. The beads were then resuspended in 2x RNA Binding Buffer and added to the RNA samples. The samples were heated at 65°C for 5 min then cooled to 4°C. This was then resuspended, incubated at room temperature for 5min, resuspended a second time and incubated a second time. The samples were placed on a magnetic rack and the supernatant discarded. Each sample was washed twice with Wash Buffer, then resuspended in 50 μl Tris Buffer and mixed. The samples were heated at 80°C for 2 min then cooled to 25°C and diluted with 2x RNA Binding Buffer. Samples were subsequently incubated at room temperature for 5 min, resuspended, and incubated again. These were then placed on the magnetic rack, and the supernatant discarded. Samples were washed with Wash Buffer, and all supernatant thoroughly removed and discarded. mRNA was eluted from the beads by adding 17 μl 10 mM Tris pH7.8 and incubating at 80°C for 2 min then held at 25°C. Samples were placed on the magnetic rack, and the supernatant transferred into a fresh tube. RNA concentration was measured by Agilent Bioanalyzer RNA chip and Thermo Fisher Qubit RNA HS (high sensitivity) Assay kit.

### RNA library preparation for Illumina sequencing

50 ng poly(A)-selected mRNA was incubated with 5X NEBNext First Strand Synthesis Reaction Buffer and 1 μl NEBNext Random Primers in 10 μl total volume. The samples were incubated for 15min at 94°C, then cooled on ice. Added to this was 0.5μl Murine RNase Inhibitor, 0.1μg Actinomycin D, 1μl Protoscript II Reverse Transcriptase and 8.5μl nuclease-free H _2_O. The samples were then incubated at 25°C for 10 min, 42°C for 15 min, then 70°C for 15 min. 10x Second Strand Synthesis buffer, 4 μl Second Strand Synthesis Enzyme mix and H _2_O were added to the samples to a final volume of 80μl, and the tubes were incubated in a thermocycler for 1 h at 16°C. Samples were then purified using a QIAquick PCR purification kit as follows: 5 volumes of PB buffer were added to 1 volume of PCR reaction and mixed. The mixture was applied to a QIAquick column centrifuged at 16,250 g for 1min. The flow-through was discarded. 750 μl PE buffer was then added to the column and the column centrifuged as above, then the flow-through discarded. The column was then transferred to a fresh 1.5 ml Eppendorf and left with lid open for 5 min. This was centrifuged for 2min. The column was then transferred to a fresh 1.5 ml Eppendorf, 58 μl 10 mM Tris pH7.8 pipetted into the centre of the column and the column left to stand for 1 min. Samples were eluted by centrifugation for 1min and stored at -20°C overnight.

10x NEBNext End Repair Reaction Buffer and 3 μl NEBNext End Prep Enzyme Mix were added to the thawed purified double stranded cDNA. The samples were incubated at 25°C then 65°C for 30 min each, before cooling to 4°C. 15 μl Blunt/TA Ligase Master Mix and 1.5μM NEBNext Multiplex Adapter were added directly to the End Prep reaction mix along with nuclease-free water to make a total volume of 83.5 μl. Samples were mixed and incubated for 15 min at 20°C. The reactions were then purified using the QIAquick PCR purification kit as above. Samples were eluted in 20 μl 10 mM Tris pH7.8.

A total of 3 μl NEBNext USER enzyme, NEBNext Q5 2x Hot Start HiFi PCR Master Mix, 2.5 μl Universal PCR Primer and one 2.5 μl Index Primer per PCR reaction (1-9 for samples 1-9) were added to the 20 μl cDNA and mixed. Samples were subjected to PCR by the following method: 37°C for 15min, [98°C for 30 s, 98°C for 10 s, by 65°C for 75 s] for 12 cycles, 65°C for 5 min then held at 4°C. The PCR reactions were then purified using the QIAquick PCR purification kit. Samples were eluted in 18 μl in 10 mM Tris pH7.8. Each sample was purified on a 3% w/v MetaPhor agarose 1X TBE gel (Fisher Scientific 10X Tris/Borate/EDTA solution) with a Fisher Scientific exACTGene 50 bp Mini ladder and 1:10,000 Invitrogen SYBR safe DNA gel stain, until bromophenol blue had migrated the length of the gel. The band ranging between 150–200 bp was extracted. This was purified using the QIAquick Gel Extraction Kit as follows: 6 volumes of QG buffer were added to 1 volume of gel. This mix was incubated at 50°C for 10 min, vortexing occasionally. 1 volume of isopropanol was added and the mix inverted. This mix was applied to a MinElute column and centrifuged at 16,250 g for 1 min. The flow-through was discarded, and the remainder of the mix applied and centrifuged as above. The column was then washed with 500 μl QG buffer and the column centrifuged again, with flow-through discarded. 750μl PE buffer was then used to wash the column, followed by centrifugation of the column and transfer to a fresh 1.5ml Eppendorf. The column was left to dry for 5min, then centrifuged for 3 min. In a fresh 1.5 ml Eppendorf, 16 μl 10 mM Tris pH7.8 was added to the centre of the column, left to incubate for 2 min, then the cDNA eluted by a 1 min centrifugation. The quality of library was assessed by Agilent Bioanalyzer DNA chip and Thermo Fisher Qubit DNA HS Assay kit.

### Quantitative PCR for mRNAs

RNA was measured by Thermo Scientific NanoDrop Spectrophotometer and DNase-treated as per the Ambion Turbo DNase protocol as follows: 10X TURBO DNase buffer and 2U TURBO DNase was added to 10μg RNA diluted in 45μl H2O. This was incubated at 37°C for 30 min, before adding 10X DNase Inactivation Reagent. This mix was incubated at room temperature for 5 min, mixing occasionally. The mix was then centrifuged at 9,250 *g* for 1.5 min, before transferring the supernatant to a fresh tube. 0.5 μg of this was then used in RETROscript’s Reverse Transcriptase kit. Random decamer primers or oligo(dT) primers were added to a final concentration of 5 μM and nuclease-free H2O added to a final volume of 12 μl. The sample was mixed and heated at 80°C for 3 min, then incubated briefly on ice. 10X RT (reverse transcription) buffer was added, together with 4 μl dNTP mix (2.5mM per dNTP), 10 U RNase Inhibitor, and 100 U Reverse Transcriptase. The sample was mixed and incubated at 42°C for 1 h, followed by 10 min at 92°C. The RT sample was then diluted to either 1 ng μl ^-1^ or 0.1 ng μl ^-1^. 4 μl of this was mixed with 6 μl of: Takara Bio SYBR Premier Ex Taq 2 X mix, 50 X ROX reference dye and 10 μM of forward and reverse primers (final concentrations 1 X, 1 X and 0.2 μM, respectively). This was then amplified by qPCR by the Agilent Stratagene Mx3005P, using the following method for SYBR Green with dissociation curve: 95°C for 1 min, [95°C for 15 s, 55°C for 15 s, and 72°C for 15 s] for 40 cycles, followed by 95°C for 1 min, 55°C for 30 s and 95°C for 30 s. Primer sets were tested with a standard curve of genomic DNA before use with samples, and each primer set tested with a no-template mix in the qPCR. Each sample was run with a no-RT control of the same concentration to ensure DNA contamination was minimal. For a subset of qPCRs, samples were DNase-treated, then poly(A)+ selected. RNA from this was then used in the RT reaction, using random decamer primers or oligo(dT) primers. qPCR was carried out as described above.

### Analysis of qPCR data for mRNAs

To determine the 2-∆∆Ct for each experiment technical replicates for both the test gene and *TAF10* were normalized to the corresponding replicate of added *Schizosaccharomyces pombe ACT1*, to account for experimental variance between samples. The test gene was then normalized to the non-target, housekeeping gene *TAF10*, and the average of each biological replicate taken. These were then normalized to the average of each BY4741 biological replicate, and a two-tailed homoscedastic t-test applied (two samples, equal variance). The difference between the Ct (threshold cycle) of *TAF10*) in the deletion strain was calculated, as was the difference between the Ct of the test gene and the housekeeping gene in the WT strain. The latter value was subtracted from the former to give the ∆∆Ct value. The exponential of –∆∆Ct gave the relative expression of the test gene in the deletion strain. The average of 2-∆∆Ct replicates for each gene was taken to determine fold change (FC). A one-sample t-test was performed to test for statistical significance.

## Bioinformatics

### Analysis of yeast CLASH data

Raw sequences preprocessed prior to alignment using hyb version 0.0 ( Travis *et al.*, 2014) running the hyb preprocess command with standard parameters. The preprocessed data were aligned to a custom database containing unspliced genes (with snoRNA genes extended by 20 bp in each direction and masked out of the genes in which they are contained where appropriate). A custom database was built using reference data from ensembl release 77. Alignment was performed using the blastall command, using the standard parameters from the hyb pipeline. The aligned reads were processed using a variant of the hyb pipeline, modified slightly to extract snoRNA hybrids rather than microRNA hybrids preferentially. Downstream analysis was performed on reproducible hybrids (in which both fragments were found to overlap in two or more hybrids) with a predicted folding energy of -12dG or below. The analysis was performed using hybtools version 0.3 ( Dunn-Davies, 2018). Reference data for the analysis of yeast methylation sites were obtained from the snoPY database ( Yoshihama *et al.*, 2013).

### snoRNA alignment

NCBI BLASTn alignments of snoRNAs were performed using parameters: Expect threshold; 10: Word size; 11; Match/mismatch; 2, -3: Gap costs; existence 5, extension 2.

### Analysis of RNA-Seq data

Raw reads were reverse complemented using FASTX-Toolkit version 0.0.14 ( Gordon, 2010) and trimmed using Trimmomatic version 0.32 ( Bolger *et al.*, 2014) , to remove Illumina adapters.

For the U3 depletion experiments, transcript level quantification was then performed using kallisto version 0.43.1 ( Bray *et al.*, 2016), using a *Saccharomyces cerevisiae* transcript database built from Ensembl release 77. Finally, differential expression was quantified using sleuth version 0.29.0 ( Pimentel *et al.*, 2017).

In the case of the snR4 depletion experiments, the trimmed reads were mapped against the *Saccharomyces cerevisiae* genome (Ensembl release 77) using STAR version 2.4.2 ( Dobin *et al.,* 2013). Gene level read counts were then obtained using htseq-count, from HTSeq version 0.9.1 ( Anders *et al*., 2015), and differential expression analysis was performed using DESeq2 version 1.11 ( Love *et al.*, 2014).

The results of the differential expression analyses of all of the depletion experiments were compared with mRNA targets of snoRNAs from the CLASH data.

### Statistical analysis

Statistical tests to determine whether CLASH targets were significantly over-represented among differentially expressed genes in the RNA-Seq analysis were carried out using the chisq.test and fisher.test functions from the stats package in R version 3.4.0.

### Sequence data

All sequence data from this study have been submitted to the NCBI Gene Expression Omnibus under accession numbers GSE114680 and GSE118369.

## Results

### snoRNA-rRNA interactions

**Figure 1.**
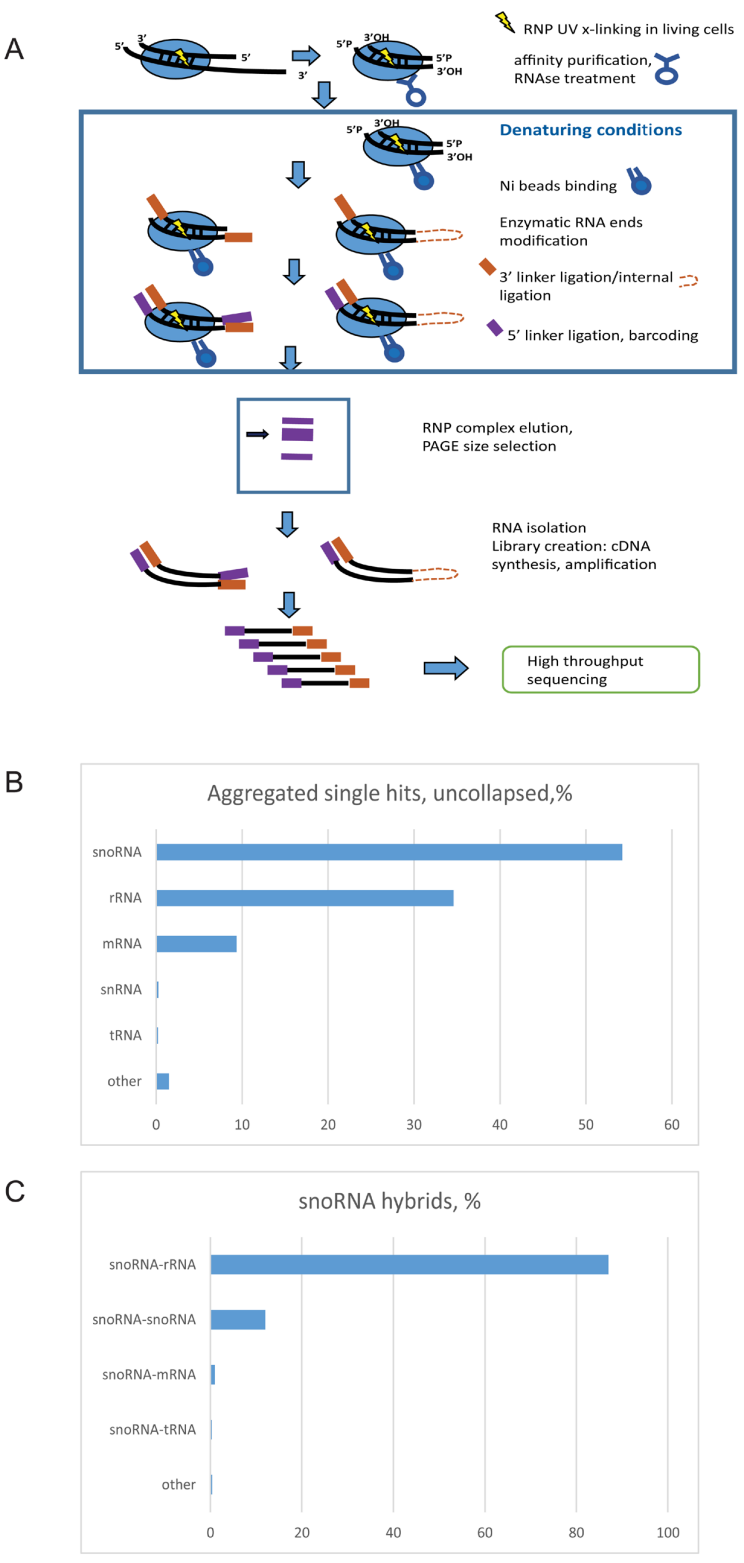
CLASH outline and results. ( **A**) Scheme of the CLASH technique. Live cells were UV irradiated, crosslinked RNA–protein complexes extracted and affinity purified. In FLASH, purified complexes were crosslinked to the beads and subsequent steps carried out with denaturing washes. ( **B**) Distribution of protein crosslinking sites by RNA type. ( **C**) Distribution of chimeric reads involving snoRNAs, identified by CLASH and sorted by target RNA type.

The construction of yeast strains expressing tagged forms of the snoRNP proteins Nop1, Nop56 or Nop58 under the control of the endogenous promoters has previously been reported ( Granneman *et al.*, 2009). The tagged constructs are the only form of these proteins in the cell and support wild-type growth, showing them to be functional. To identify potential novel snoRNA interactions we applied the CLASH technique ( Helwak *et al*., 2013; Kudla *et al*., 2011). This involves crosslinking of RNA complexes with tagged proteins by UV in living cells, affinity purification of the RNP complexes under stringent conditions, ligation of linker adaptors in parallel with internal ligation of captured RNA fragments base paired to each other, isolation of RNA, including RNA hybrids, followed by reverse transcription and high-throughput sequencing of cDNA libraries ( Figure 1A). We performed total of 26 independent experiments using protein-tagged Nop1, Nop56 or Nop58 as bait in *S. cerevisiae*.

Analyses of single hits for yeast Nop1 during growth in glucose medium showed that snoRNAs were most frequently recovered, both for pooled reads ( Figure 1B) and individual proteins ( Supplementary Figure 1A–C) followed by rRNA and mRNA. In contrast, for Nop56 and Nop58, rRNA sites were most frequently recovered. Recovered sequences that could be confidently mapped to two distinct regions of the genome (see Methods) were regarded as representing chimeric cDNAs resulting from RNA-RNA ligation.

**Figure 2.**
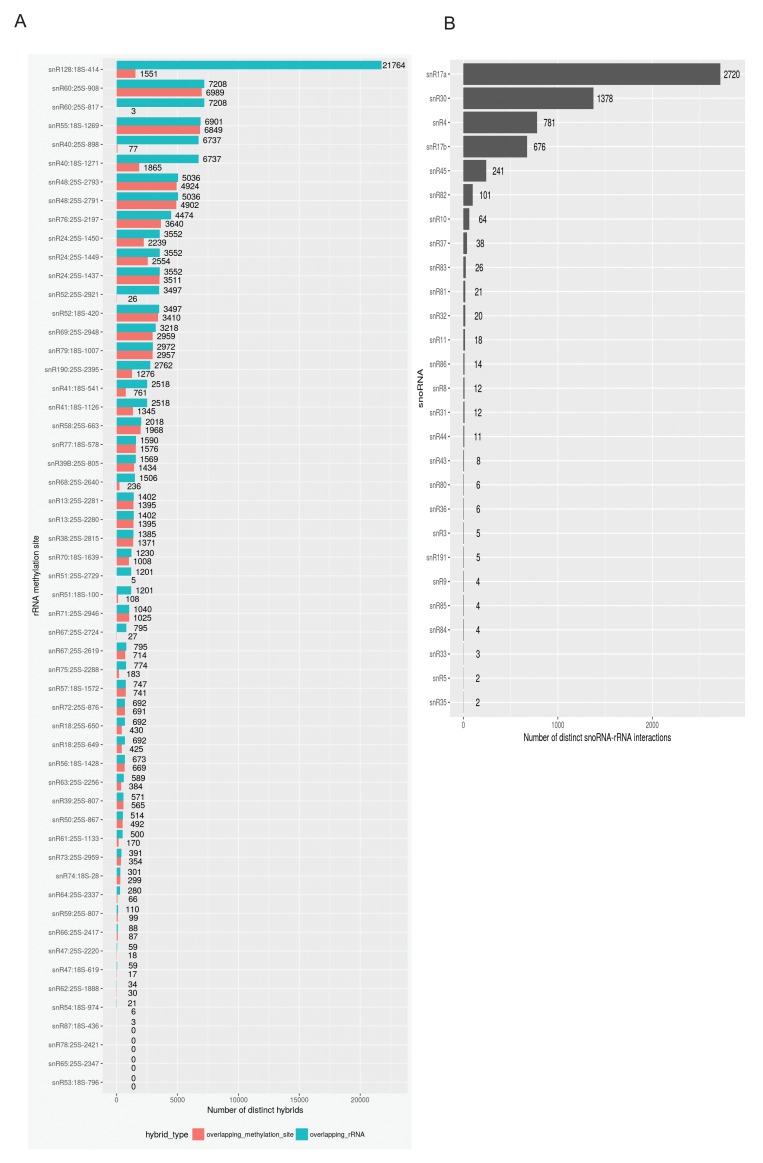
Numbers of snoRNA-rRNA interactions at sites of 2’- *O*-methylation. ( **A**) All non-identical snoRNA-rRNA interactions overlapping each site of 2’- *O*-methylation in rRNA were tabulated. For each methylation site, numbers are indicated separately for snoRNA interactions overlapping the methylation site (upper green bar) and interactions predicted to guide methylation (lower red bars). ( **B**) All non-identical snoRNA-rRNA interactions not overlapping 2’- *O*-methylation sites in the rRNA were tabulated. Note that none of the top scoring snoRNAs are expected to direct methylation.

In total, we recovered 241,420 distinct hybrids in yeast, of which 123,642 were snoRNA-rRNA hybrids. Within our dataset, we found snoRNA-rRNA hybrids for all of the 43 C/D box snoRNAs reported to methylate yeast rRNA in the snOPY database ( Yoshihama *et al.*, 2013). For 42 of these snoRNAs (all except snR65), hybrids were present in the dataset that overlapped each of the reported methylation sites in rRNA associated with the relevant snoRNA ( Figure 2). For comparison, we reanalyzed our previously published data ( Kudla *et al.*, 2011) using the same pipeline ( Supplementary Figure 2).

*In silico* folding of the hybrid sequences using the ViennaRNA package ( Lorenz *et al*., 2011) was used to predict the stability of the base-paired interaction that gave rise to the hybrid. In order to identify stable, base-paired interactions, only chimeric sequences with a predicted ∆G of less than -12 kcal mol ^-1^ were retained for analysis. Non-identical chimeric sequences, or sequences recovered from different analyses, in which both segments overlapped were regarded as demonstrating independent recovery of the same interaction. Only interactions supported by at least two independent sequences with a predicted ∆G of less than -12 kcal mol ^-1^ were considered stable and reproducible, and further analyzed. A total of 190,597 hybrids passed these filters, of which 117,047 were snoRNA-rRNA hybrids.

From the set of reproducible hybrids, 87% of the snoRNA-interacting sequences were mapped to the rDNA, 12% to another snoRNA, 1% to mRNAs and 0.4% to other RNA species ( Figure 1C). It is notable that some highly abundant RNA species were recovered at low levels, particularly tRNAs (0.2% of total hybrids or 0.3% of snoRNA hybrids before filtering), supporting the specificity of the interactions. The predominant recovery of snoRNA-rRNA interactions is in agreement with the known function of snoRNAs in ribosome synthesis.

**Figure 3.**
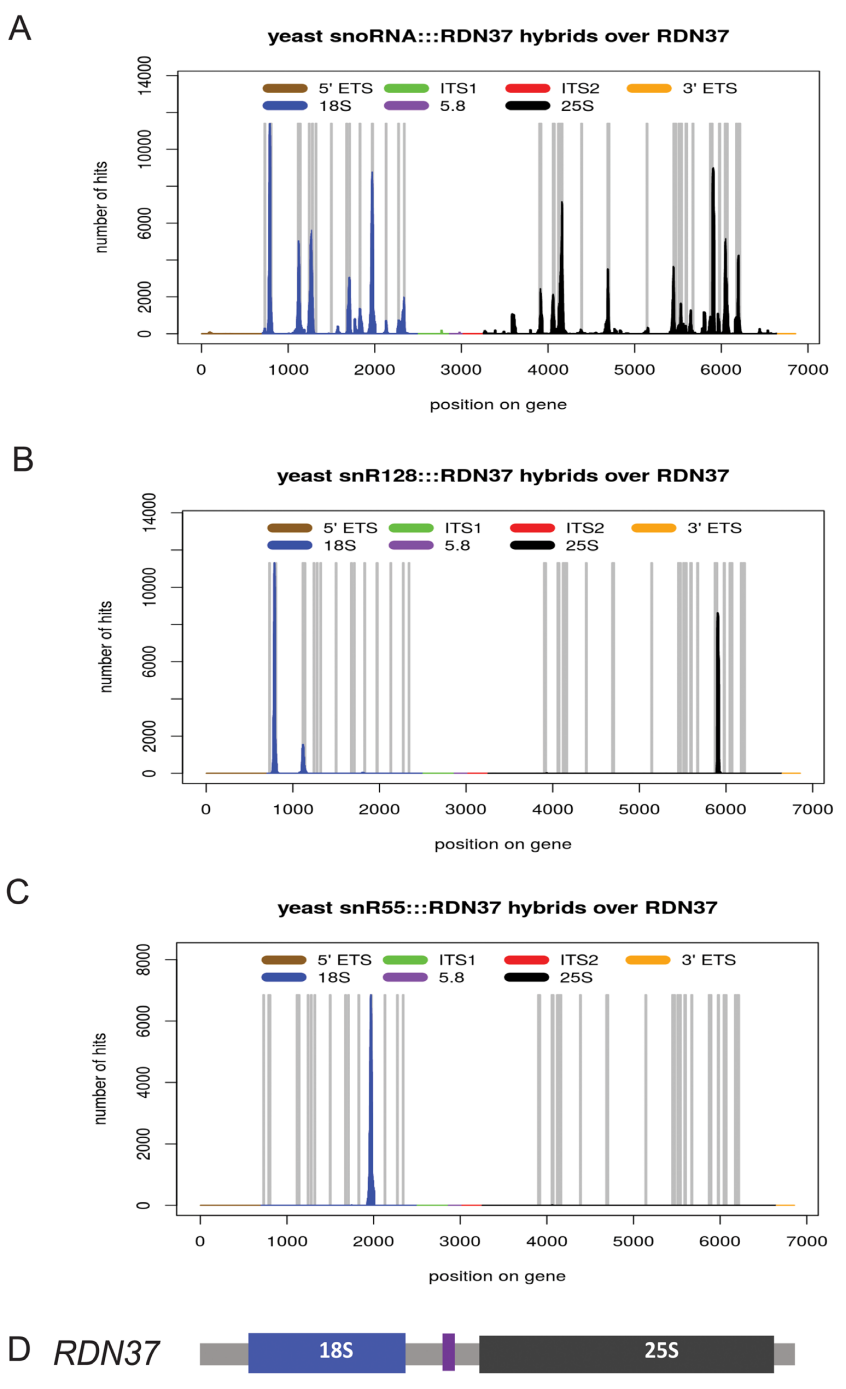
Distribution of snoRNA interactions on the rRNAs. Chimeric cDNAs between snoRNA sequences and the pre-rRNAs were identified by mapping to the rDNA locus *RDN37* and the number of hybrid hits for each location was plotted. The peaks were located preferentially in the regions of known methylation sites and structural interactions. ( **A**) All snoRNA-rRNA chimeras mapped to the rDNA. Vertical bars in grey indicate the sites of rRNA 2’- *O*-methylation. ( **B**) Distribution chimeric reads involving snR128 (U14). ( **C**) Distribution chimeric reads involving snR55. ( **D**) Schematic of the rDNA locus, indicating the locations of the rRNA genes.

On the 35S pre-rRNA sequence, 116,611 stable, reproducible chimeras between snoRNAs and the 18S, 5.8S or 25S rRNA were mapped to 601 high confidence interaction sites. Inspection of the locations of snoRNA-rRNA hybrids showed colocalization with snoRNA-directed methylation sites along the mature 18S and 25S rRNA (shown in grey in Figure 3A). Low signals were seen over the transcribed spacer regions and 5.8S rRNA, which lack methylation sites. Comparing to the known sites of rRNA methylation, we identified high-confidence, cognate snoRNA-rRNA hybrids that overlapped 51 of the 55 reported methylation sites, involving 40 of the 43 methylation guide C/D box snoRNAs; shown for snR128 (U14) and snR55 ( Figure 3B, C). In total, 10 yeast snoRNAs gave rise to 66% of reproducible rRNA hybrids; snR128 (U14), snR60, snR55, snR40, snR48, snR76, snR24, snR17 (U3), and snR79. Reported methylation guide snoRNAs for which we did not find stable, reproducible snoRNA-rRNA hybrids were snR53, snR65, and snR78 ( Figure 2A and Supplementary Table 2). A total of 53% of hybrids overlapping methylation site showed interaction pattern that supports methylation of the target nucleotide in rRNA ( Supplementary Table 2).

We also observed many examples of interactions in which methylation sites are bound by non-cognate snoRNAs that would be predicted to block methylation guide function. It is possible that competition between snoRNAs can exert additional level of regulation of ribosome modification ( Supplementary Table 2). All methylation sites in the yeast rRNA have been confidently mapped ( Taoka *et al*., 2016; Yang *et al*., 2016), making it unlikely that blocking interactions correspond to unknown methylation sites, at least under standard lab growth conditions.

Under conditions of reduced growth, the rate of ribosome synthesis is also reduced. To assess whether this was associated with altered snoRNA interactions, crosslinking was preformed following transfer of cells from glucose medium to medium containing 2% ethanol + 2% glycerol as sole carbon sources for 20 min. Some snoRNAs indeed displayed different binding pattern under different growth conditions (shown for snR4 in Supplementary Figure 3). Values for the growth of strains in different growth media is available on figshare ( Dudnakova *et al*., 2018).

A small number of yeast box C/D snoRNAs are not implicated in rRNA methylation and hybrids with the rRNA were also recovered for these species ( Figure 3B). These are U3 (encoded by the genes *SNR17A* and *SNR17B*), snR4 and snR45. U14 (encoded by *SNR128*) has two characterized sites of interaction; the sequence flanking box D directs methylation, while base-pairing of the sequence flanking box D’ is required for early pre-rRNA processing. We recovered snR17(U3) interactions with 18S as previously described ( Kudla *et al*., 2011). U3 interacted with pseudoknot region of rRNA via its region located immediately upstream of the D box sequence and partially in the helix 3 ( Supplementary Figure 4). Earlier studies on U3 snoRNA structure and function showed that conserved boxes A, A’, B, C, C’ and D are important for U3 function in rRNA maturation. In contrast, helixes 2, 3, 4 were reported to be non-essential for U3 stability and function, although the essential protein Rrp9 binds to helixes 2 and 3 ( Samarsky & Fournier, 1998; Venema *et al.*, 2000).

**Figure 4.**
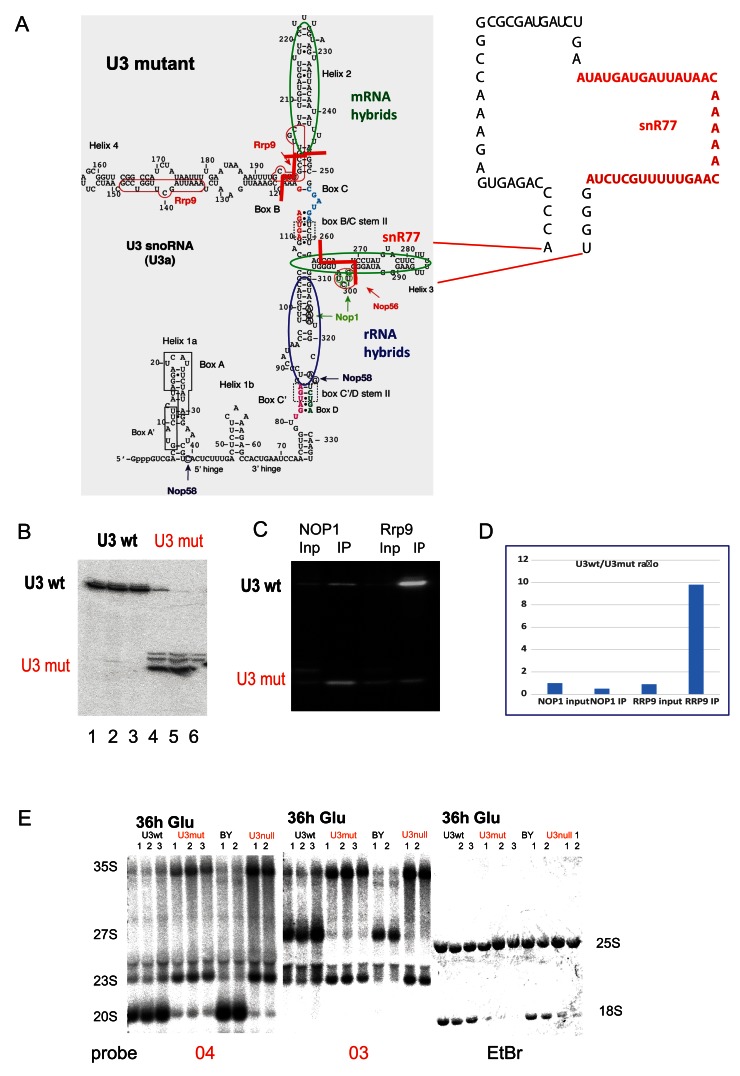
Effects of U3 mutations on pre-rRNA processing. ( **A**) Left: Predicted structure of U3A. Regions forming hybrids with the rRNA identified by CLASH are circled in blue. Regions forming hybrids with mRNAs are circled in green. End points of deletions are indicated with red lines. Major crosslinking sites for snoRNP proteins are indicated with arrows and nucleotides are circled. Right: Structure of U3 deletion construct. Regions deleted or substituted with the snR77 sequence are indicated with dotted lines. ( **B**) Northern blot showing expression of the wild type (U3 wt) and the truncated U3 (U3 mut). Lanes 1–3 and 4–6 show biological triplicates. ( **C**) RNA IP northern blot from the strains expressing either TAP-Nop1 or TAP-Rrp9 and mutant U3. ( **D**) Quantification of RNA IP. Signal densitometry of the selected lanes were measured with AIDA Image Analyzer v.4.15 densitometry software and plotted. ( **E**) Northern analysis of pre-rRNA processing following transfer of the U3mutant strain to glucose medium for 36 h. U3 strains have endogenous U3 expression under *P _GAL_* control, complemented by plasmid expression of wild type U3 (U3 wt lane) or the truncated mutant (U3mut lanes). As controls, the parental strain (BY) and the non-complemented (U3null lanes) strains are shown. Lanes 1–3 or 1 and 2 for each sample show biological replicates. Site A2 is the 3’ end of the 20S pre-rRNA and the 5’ end of 27SA and 27SB pre-rRNAs. Probe 03 is located in the ITS1 region of the pre-rRNA, 3’ to cleavage site A2. Probe 04 is located in the ITS1 region 5’ to site A2.

We recovered multiple hybrids between these helixes and the 35S pre-rRNA ( Supplementary Figure 4 and Supplementary Table 2). The snoRNAs U3 and U3b are functionally redundant but essential for growth and pre-rRNA processing. To allow functional assays of U3, we deleted the *SNR17B* genetic locus and placed the expression of *SNR17A* under the control of a repressible *P _GAL_* promoter. For complementation tests, we generated a plasmid expressing the truncated mutant U3 under the endogenous promoter. Helixes 2 and 4 of wild-type U3 snoRNA were deleted and the region of mRNA interaction in the helix 3 was replaced with snR77 mRNA binding sequence ( Figure 4A). Analysis of a strain lacking the cluster genes, *SNR72-SNR78* (see Materials and Methods), showed no clear alterations in mRNA levels, making snR77 appear a suitable sequence donor. U3mut was expressed under the control of the native *P _SNR17A_* promoter in a strain carrying *P _GAL_::SNR17A, snr17b∆*, which conditionally expresses U3a under control of a galactose-inducible promoter and lacks U3b. In galactose medium, mutant U3m was stable and expressed at the levels comparable with endogenous U3a ( Figure 4B and Dudnakova *et al*., 2018). Following transfer to glucose to deplete endogenous U3, the strain was inviable ( Supplementary Figure 4) in contrast to the previous report ( Samarsky & Fournier, 1998). Maturation of 18S rRNA was inhibited, with accumulation of 23S and 27SA2 pre-rRNA ( Figure 4E). This phenotype reflects inhibition of pre-rRNA cleavage at sites A _0_, A _1_ and A _2_, and is characteristic of the effects of loss of U3 or U3-associated proteins, including Rrp9 ( Venema *et al.*, 2000; Zhang *et al.*, 2013). Rrp9 was shown to interact with U3 in the helix 2 and 4 regions, and box C was shown to be important for Rrp9 binding. We propose that U3 binding is needed to tether Rrp9 to the pre-ribosome during maturation. This suggestion was supported by RNA-immunoprecipitation ( Figure 4C, D) showing that Rrp9 was unable to bind the mutant U3, whereas Nop1 binding was similar for mutant and wild type U3.

### Targets for snR4 and snR45

The non-essential snoRNAs snR4 and snR45 were not known to play any role in ribosome synthesis when these analyses commenced. Strains carrying *snr4∆* or *snr45∆* showed no detectable growth defect relative to the isogenic wild-type ( Supplementary Figure 5A and Dudnakova *et al*., 2018). As snR4 and snR45 were the only snoRNAs whose deletion did not impact on ribosome synthesis, it seemed feasible that they might have redundant functions, and we therefore generated double mutant strains carrying *HISMX6-P _GAL1_::SNR45 snr4∆* and *HISMX6-PGAL1-SNR4 snr45∆*. However, growth of the double mutant strain on glucose medium to deplete the GAL-regulated snoRNA did not confer a detectable growth phenotype ( Supplementary Figure 5A).

To test for evolutionary conservation, the *SNR4* and *SNR45* sequences were analyzed using NCBI BLAST *,* optimizing for somewhat dissimilar sequences (blastn). A block of ~200nt was identified around the synonymous regions from distantly related fungal genomes and aligned using the MultAlin online alignment tool ( Corpet, 1988). *SNR45* shows regions of conservation to fungal homologues and human U13 (SNORD13) ( Supplementary Figure 5). Conservation was high over the 5’ region, box C (nts 28-34), box D’ (nts 97-100), box C’ (nts 108-114) and box D (nts 193-196). However, the regions flanking box D and D’, which would be the expected locations of the methylation guide were poorly conserved, whereas a well-conserved region was identified, ** spanning nts 140-151 ( Supplementary Figure 6).

Comparison of *SNR4* to fungal homologues ( Supplementary Figure 6) showed around box C (nts 15-21). Box C’ is also highly conserved (nts 123-129) but no clear D’ box, was identified. Box D is well conserved at nucleotides 184-187. A further well-conserved region was noted (nts 146-155), which does not correspond to known structural features or the expected location of a methylation guide sequence.

While this work was underway, it was reported that snR4 and snR45 direct 18S rRNA acetylation, at positions m5C1280 and m5C1773, respectively ( Sharma *et al.*, 2015; Sharma *et al.*, 2017). Analysis of the CLASH data identified hybrids between snR4 and snR45 and sequences flanking the acetylated residues. For snR45, we recovered two snR45 hybrids with the sequence flanking ac ^4^C1773 ( Supplementary Figure 5). The nucleotides involved in base-pairing correspond to the region high conservation noted in snR45 homologues. Notably, in analyses of human snoRNAs (manuscript in preparation), we identified a homologous interaction between U13 and the corresponding methylation site Ac1842 ( Supplementary Figure 5).

Supplementary Figure 6 shows two examples of the 17 hybrids found between snR4 and this the region of 18S rRNA upstream of the ac ^4^C1280 (∆G -13.8). The nucleotides involved in base-pairing corresponded to the region of high conservation between fungal snR4 homologues. Even using this region of conservation, no clear human homologue could be identified for snR4.

### Mtr4 is associated with non-cognate snoRNA interactions

**Figure 5.**
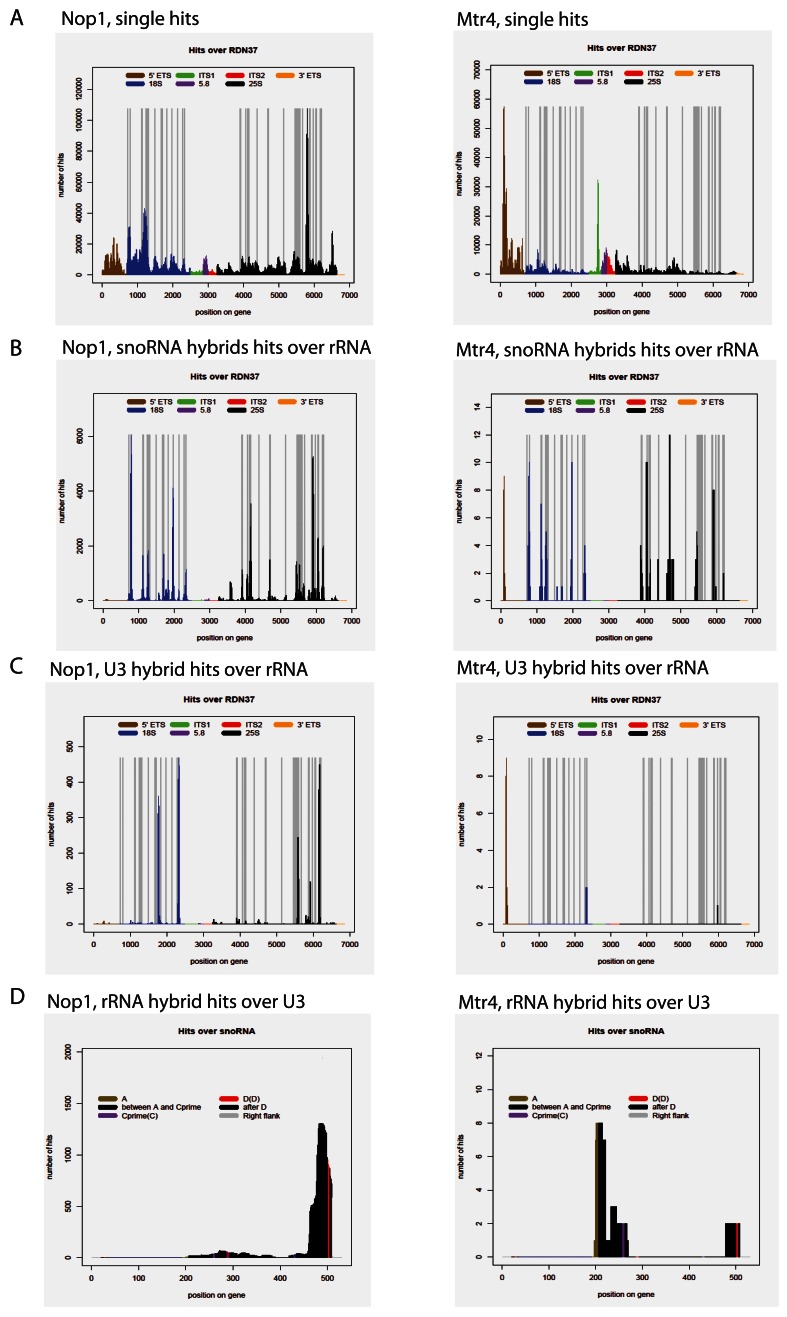
Comparison of snoRNA-rRNA interactions obtained in NOP1 and MTR4 CLASH. NOP1 CLASH data shown on the left, MTR4 CLASH data shown on the right. ( **A**) Single hits mapped to the rDNA. ( **B**) All snoRNA chimeras mapped to the rDNA. Vertical bars in grey indicate the sites of rRNA 2’- *O*-methylation. ( **C**) Distribution chimeric reads involving snR17A/snR17B (U3). ( **D**) Distribution of RDN37 chimeric reads over the *SNR17A* gene.

Many apparently stable (i.e. ∆G < -12 kcal mol ^-1^) snoRNA interactions were recovered at rRNA sites not predicted to guide methylation. Mtr4 is a 3’-5’ RNA helicase and cofactor for the nuclear exosome complex, which appeared to be candidate factor that might participate in displacing snoRNAs from the pre-rRNA, particularly those bound at inappropriate locations. We therefore generated CLASH data for Mtr4-HTP ( Delan-Forino *et al.*, 2017). Single hits from Mtr4 CLASH demonstrated the binding profile consistent with the known role of Mtr4 in pre-rRNA processing and previous results ( Figure 5A) ( Delan-Forino *et al.*, 2017). Mapping snoRNA hybrid hits on the pre-rRNA showed that most chimeras recovered overlapped known methylation sites ( Figure 5B). Strikingly, however, when analyzed individually, the snoRNA binding profiles predominately do not show the expected binding pattern for methylation guide interactions. Chimeras recovered with Mtr4 and predicted to correctly guide methylation constituted only 7.2% of all hybrids overlapping methylation sites ( Figure 5C, D), in contrast to 53% for Nop1, Nop56 and Nop58.

We propose that snoRNA docking at non-cognate sites is common, but is specifically relieved by the activity of Mtr4.

### snoRNA-snoRNA interactions

Numerous stable, reproducible snoRNA-snoRNA hybrids (15,736) were identified. The majority represented snoRNA intermolecular stems, which reveal potential information on snoRNA secondary structure. In addition, 2,734 hybrids represented interactions between different box C/D snoRNAs, including 78 different snoRNA-snoRNA combinations.

**Figure 6.**
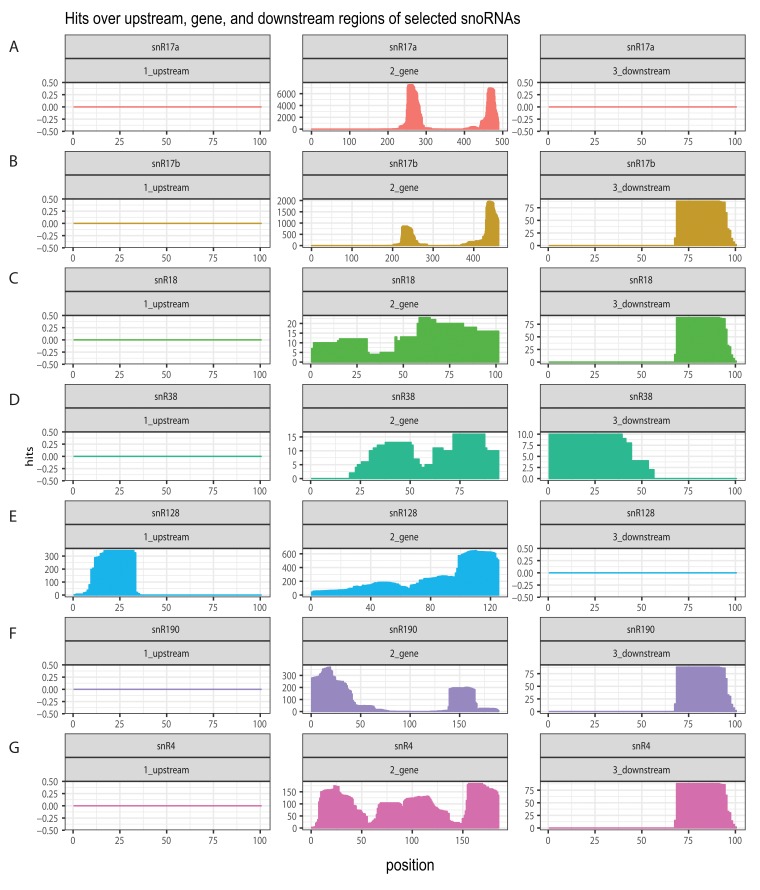
Distribution of snoRNA hits over the corresponding snoRNA genetic locus. Chimeric snoRNA-snoRNA cDNAs were mapped onto the genetic locus containing the snoRNA gene with 100bp upstream and downstream regions; the number of hybrid hits for each position was plotted. ( **A**) snR17a; ( **B**) snR17B; ( **C**) snR18; ( **D**) snR38; ( **E**) snR128 (U14); ( **F**) snR190; ( **G**) snR4.

Interactions were also identified between mature snoRNA sequences and the 5’ and 3’ flanking regions. These presumably occur within the snoRNA precursors and potentially function during snoRNP biogenesis ( Figure 6). Notably, the region 70 nt 3’ to the mature snoRNA was a strongly favored target, both for presumed interactions in *cis* within the pre-snoRNA and in *trans* with other snoRNAs. These positions potentially represent important regulatory sites that are presented for RNA binding. We noted that the pattern was different for snR128, potentially related to its production from a bicistronic precursor with snR190. Based on these finding we speculate the existence of regulatory loops, particularly in snoRNA biogenesis.

### snoRNA-mRNA interactions

**Figure 7.**
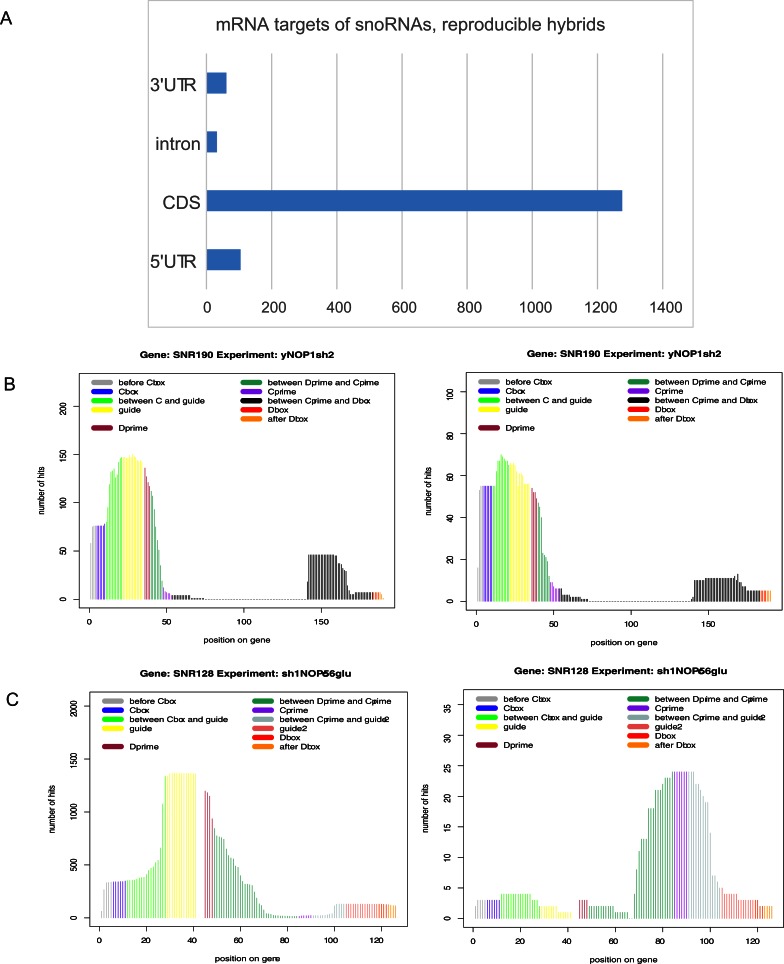
snoRNA-mRNA interactions. ( **A**) Distribution of snoRNA hits over mRNA features. Chimeric cDNAs between snoRNA sequences and the mRNA sequences were identified by mapping to the yeast genome (ensembl release 77; see Methods) followed by allocation to the annotated mRNA features. The number of snoRNA hybrids involving mRNA UTRs, exons or introns was plotted. ( **B**) Hybrid hits involving rRNA (left) and mRNA (right) hits plotted over snR190. ( **C**) Hybrid hits involving rRNA (left) and mRNA (right) hits plotted over snR128 (U14).

A potentially significant class of snoRNA chimeras involved snoRNA-mRNA interactions, which constituted 1.9% of all chimeric reads. These included 1,368 reproducible hybrids that represented 448 distinct interactions involving 39 snoRNAs and 382 mRNAs. Only hybrids with energy of interaction dG <-12 kcal mol ^-1^ and independently recovered at least twice were included in analyses. Analyzing the distribution of reproducible snoRNA interactions across gene features identified 105 sites in 5’ UTRs, 1276 sites in coding sequences, 32 sites in introns and 61 sites in 3’UTRs ( Figure 7). Five snoRNAs (snR190, snR17a/b (U3), snR128 (U14), snR76, snR40) formed 63% of the recovered mRNA hybrids. Hybrids were also frequently recovered with snR39B and the orphan snoRNAs snR4 and snR45. Notably, several snoRNAs interacted with mRNAs via regions that were distinct from the rRNA binding sites (shown for snR128 (U14) in Figure 7). The filtered list of stable, reproducible snoRNA-mRNA interactions is presented in Supplementary Table 3; the complete list of interactions and sites is given in Supplementary Table 4.

Most snoRNA-mRNA duplexes are predicted to represent “structural” interactions that do not direct RNA methylation. However, we discovered 15 snoRNA-mRNA interactions that potentially promote methylation of target mRNAs ( Supplementary Table 5).

We observed a correlation between hybrid and single mRNA hits, supporting the specificity and relevance of the hybrids ( Supplementary Figure 7; see Pearson correlation between hybrid and single hits). In contrast, there was no clear correlation between mRNA expression level and presence in snoRNA hybrids ( Supplementary Figure 7). These findings support the conclusion that the RNA-RNA chimeras recovered represent *in vivo* interactions.

### Depletion of U3

A strain carrying *P _GAL1_::SNR17A, snr17b*∆, was transferred to glucose medium for 36 h to deplete U3. Changes in mRNA abundance were assessed by RNA-seq with NEBNext kit using a RiboMinus system to deplete rRNA from the samples. Sequence reads were mapped to the yeast genome and changes were quantified using DeSeq2 and Kallisto (see Methods).

**Figure 8.**
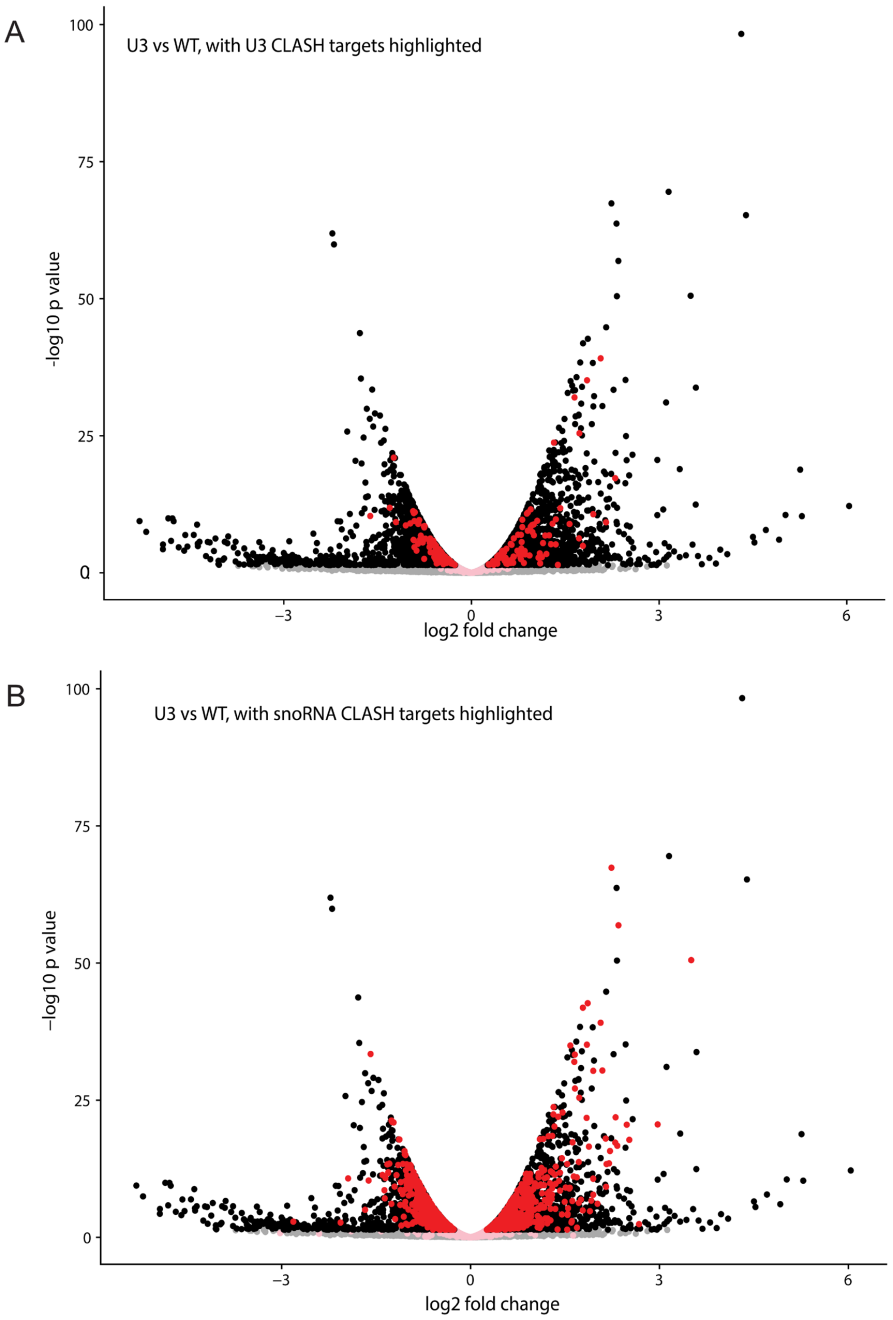
Differential RNA expression in strains expressing mutant U3. Volcano plot showing differential RNA expression in strains expressing mutant U3 compared to the wild type following growth in glucose medium for 36 h. RNA sequencing was performed following Ribominus selection. X axis shows log _2_ of normalized RNA fold change with negative values signifying decreased in RNA expression in the mutant. Y axis shows –log _10_ of the p-value. Significantly differentially expressed genes are either black (non-CLASH targets) or red (CLASH targets). ( **A**) U3 targets are highlighted in red; ( **B**) All snoRNA targets are highlighted in red.

The mRNA expression profiles obtained for U3 depletion were compared to CLASH data, to determine whether mRNAs showing altered abundance following snoRNA mutation are enriched for direct binding targets ( Figure 8A). Most mRNAs showing altered abundance were not U3 CLASH targets, although these were significantly over-represented (red dots in Figure 8A) (Chi Square Test; P<0.05). In addition, mRNA targets of snoRNAs other than U3 were also altered in the U3 strain (red dots in Figure 8B). This might reflect the finding that the mutant U3 does not support ribosome biogenesis and 35S pre-rRNA is accumulated. This might act as a sponge, leading to snoRNA sequestration on the pre-rRNA and reduced availability for mRNA binding. At the same time, snoRNAs acting in the later stages of ribosome biogenesis might show reduced rRNA binding, potentially freeing them to bind non-ribosomal targets. 

### Depletion of snR4

**Figure 9.**
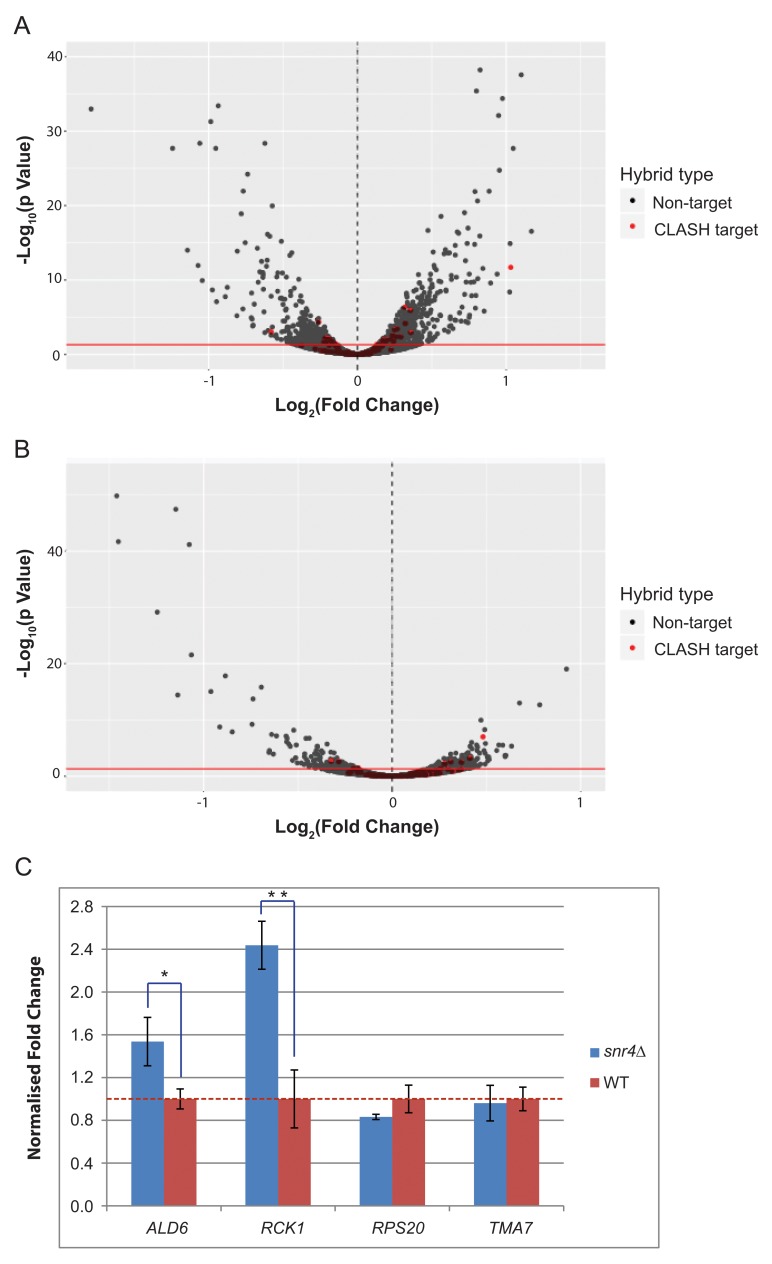
Volcano plot showing differential expression of RNAs during snoRNA deletion, using poly(A)+ selected samples. ( **A**) Differential expression of poly(A)-tailed RNAs following growth in standard conditions in *snr4∆*, compared to WT. The dotted line at 0 signifies normalized WT RNA expression level. Negative values show a decrease in RNA expression, while positive values signify an increase in RNA expression upon snoRNA deletion. Y axis shows –log10 of adjusted p value. Red line indicates p=0.05. Red dots indicate a direct target from CLASH data. ( **B**) As in ‘A’, but harvested transfer for 20 min into the media containing 2% EtOH/glycerol as the carbon source. ( **C**) RT-qPCRs showing fold change of CLASH targets following growth in standard conditions. Fold change of snR4 targets in the *snr4∆* strain compared to WT strain. All samples were normalized to S. pombe *ACT1*, then to *TAF10*, then to WT gene expression level. Red dotted line denotes relative WT expression level. RT was performed using oligo (dT) primers. One asterisk denotes p<0.05, two asterisks denote p<0.01.

To assess potential roles for snR4 in mRNA expression or stability, the *SNR4* gene was deleted from strain BY4741. The effects of *snr4∆* on mRNA levels were assessed under normal growth conditions of SD (glucose) medium at 30°C ( Figure 9A) and following stress induced by brief transfer to ice-cold PBS ( Figure 9B); the conditions used for initially reported snoRNA CLASH analyses ( Kudla *et al*., 2011). RNA abundance was assessed by RNA-seq as described for the U3 mutant strain.

As for U3, most mRNAs showing apparently altered abundance were not snR4 CLASH targets. However, there was a statistically significant (Fisher’s exact test; P <0.05) over-representation of mRNAs identified as interacting with snR4 in CLASH analyses among species showing altered accumulation in *snr4∆* strains, both during normal growth and following stress ( Figure 9A, B). RNAs showing both altered expression in *snr4∆* and recovery as snR4-mRNA hybrids were enriched in GO terms for genes involved in metabolic processes. These data suggest a possible role of snR4 in stress response, which would be consistent with differences in snR4 binding to rRNA in normal growth and stress conditions.

Selected snR4 CLASH targets that were also found to be altered in abundance by RNA-seq were further tested by RT-qPCR ( Figure 9C). Consistent with RNA-seq data, mRNAs abundances were reduced in strains lacking snR4 and showed greater effects following transfer to cold PBS than during exponential growth on glucose medium. The effects were statistically significant, but of low magnitude. Figure 9C shows the relative fold changes for the three snR4 CLASH targets and *RCK1* along the y axis, compared to WT. The red dashed line indicates a fold change of 1, showing the WT normalized expression for each gene. In standard conditions upon *SNR4* deletion, *ALD6* had a fold change of 1.54. This matched the direction of the fold change of 2.0 shown in poly(A) ^+^ selected RNA sequencing. *RCK1* showed a fold change of 2.4, which was similar to the 2.8 fold change observed in RNA sequencing. Both *ALD6* and *RCK1* had statistically significant differential expression upon *SNR4* deletion, with p values of 0.019 and 0.002, respectively. The negative control *RPS20* showed a fold change of 0.83 by qPCR, which is comparable to its fold change of 0.874 from RNA sequencing. However, the p value was 0.091, which is not significant. Similarly, *TMA7* showed a fold change of 0.860 in RNA sequencing, but had a fold change of 0.96 in qPCRs, with a p value of 0.75. Full Ct values obtained from RT-qPCR are available on figshare ( Dudnakova *et al*., 2018).

## Discussion

We report the systematic analysis of yeast snoRNA interactions, by CLASH analyses using tagged forms of the major snoRNP proteins as bait. The previously described, cognate snoRNA-rRNA pairs were recovered for most sites of yeast rRNA methylation. A further yeast snoRNA-rRNA interaction matched the consensus for methylation guide activity, but did not correspond to a reported site of methylation. In addition, we recovered a large number of snoRNA-rRNA interactions that are not predicted to direct methylation, but which would be expected to block methylation-guide interactions by other snoRNA species. Recent data point to the regulation of rRNA methylation levels in human cells (see for example ( Erales *et al.*, 2017); reviewed by ( Sloan *et al.*, 2017)) and at least one methylation and a pseudouridine site in the yeast rRNA are substoichiometric ( Buchhaupt *et al.*, 2014; Taoka *et al.*, 2016). Competitive interactions between snoRNAs offer a possible mechanism for regulating modification efficiencies.

CLASH analyses with the RNA helicase and surveillance factor Mtr4 revealed a significantly different pattern from the snoRNP proteins. Interactions between snoRNAs and rRNAs identified in association with Mtr4, predominately occurred at methylation sites. However, only a small fraction (~7%) represented cognate methylation guide interactions, compared to the ~60% cognate interactions identified with the snoRNP proteins. It was previously reported that the helicase Prp43 plays a role in unwinding cognate snoRNA-rRNA interactions ( Bohnsack *et al*., 2009). CLASH data are not available for Prp43, but the snoRNAs most frequently bound (snR51, snR72 and snR60) show only limited overlap with the major interactors for Mtr4 (snR128, snR17a, snR55, snR60, snR69). We predict that Mtr4 plays an important surveillance role in dissociating incorrect but stable snoRNA interactions. The basis of the recognition of these interactions remains unclear. However, we note that the yeast snoRNAs are of low abundance relative to the high rate of ribosome synthesis, implying rapid release following RNA modification ( Bohnsack *et al*., 2008). The extended base-paired interactions between snoRNAs and targets are expected to be very stable under physiological conditions, indicating the need for helicase activities. It seems feasible that productive, cognate snoRNA-target interactions are rapidly recognized and dissociated, whereas the non-cognate interactions are of longer duration. This could lead to backup recognition by the nuclear RNA surveillance system, of which Mtr4 is a key component.

A high prevalence of pre-snoRNA binding to other snoRNAs was observed. This could reflect compartmentalization, with enrichment of pre-snoRNAs in a subnuclear, or subnucleolar, region. An obvious possible domain would be the nucleolar body, in which snoRNA cap hypermethylation and snoRNP assembly are reported to occur ( Mouaikel *et al.*, 2002; Verheggen *et al.*, 2002). Interactions between different snoRNAs also have an obvious potential to regulate effective snoRNA availability and therefore methylation efficiency.

There are two yeast snoRNAs that have no known participation in ribosome synthesis, snR4 and snR45, and we therefore analyzed these species in more detail. Examining the conservation between distantly related fungi revealed that both *SNR4* and *SNR45* are well-conserved C/D box snoRNAs. However, both showed a pattern of conservation that was different from that of canonical box C/D snoRNAs, particularly in sequences flanking the box motifs, and each contained a similarly positioned region of high conservation that did not correspond to known structural features or the location of a methylation guide sequence. During this work, snR4 and snR45 were shown to guide C5 acetylation by the acetyl-transferase Kre33p of residues C1280 and C1773, respectively, in yeast 18S rRNA ( Sharma *et al*., 2017). Inspection of the snoRNA-rRNA hybrids identified base-pairing between the conserved regions and the sites of rRNA acetylation. More extensive base-pairing between snR4, snR45 and U13, and the 18S rRNA in the vicinity of the acetylation sites have been proposed ( Sharma *et al*., 2017). However, we did not recover hybrids corresponding to these potential interactions 

A large number of stable, reproducible snoRNA-mRNA interactions were also identified. It is currently unclear whether any of these mRNAs are actually targets for methylation, or the subcellular location of the interactions. snoRNA-mRNA interactions in the nucleolus are possible, but in metazoans snoRNAs and snRNAs undergo modification in the Cajal bodies. In yeast, snoRNA modification takes place in nucleolar bodies, which appears to be a potential site for snoRNA-mRNA binding. It is also worth noting that, although the nucleolus appears to be a stable structure when visualized in microscopy, or indeed when isolated from the cell, this impression is actually quite misleading. Nucleolar components have long been known to exchange rapidly with free cytoplasmic pools, a phenomenon that now seems likely to be due to the phase-separated nature of the nucleolus. Thus we cannot exclude the possibility that snoRNP-mRNA interactions take place in the nucleoplasm.

We observed that the absence of snR4 or mutation of U3 was associated with altered abundances of relatively small numbers of mRNA species. RNAs that were also identified as CLASH targets were statistically over-represented among mRNAs with altered abundance. Loss of snR4 was preferentially associated with decreased levels of target RNAs, potentially indicating a role in mRNA stabilization. A potential explanation might be that the snoRNP competes with one of more pre-mRNA binding protein(s) that protect the pre-mRNA and/or promote export. However, we cannot exclude the possibility that the snoRNP interacts with the nuclear RNA surveillance machinery to accelerate or retard degradation. In contrast, both increased and decreased levels were observed among U3 target mRNAs. U3 depletion was also associated with altered abundance of other snoRNAs. This could reflect either direct interactions or via the stalled pre-rRNA processing which in turn changed the levels of their target mRNAs too. These observations suggest the evolution of a fast, regulatory mechanism required for stress response during sudden changes in growth conditions.

## Data availability

All sequence data from this study have been submitted to the NCBI Gene Expression Omnibus. Sequence data from the CLASH experiments are available under accession number GSE114680: http://identifiers.org/geo/GSE114680. Sequence data from U3 and snR4 depletion experiments are available under accession number GSE118369: http://identifiers.org/geo/GSE118369

Data generated through the identification of potential novel RNA targets for box C/D snoRNAs in budding yeast are available on figshare: https://doi.org/10.6084/m9.figshare.6984971 ( Dudnakova *et al*., 2018).

## Supplementary material

**Supplementary Figure 1. Distribution of protein crosslinking sites by RNA type.**

(A) Nop1 CLASH single hits; (B) Nop56 CLASH single hits; (C) Nop58 CLASH single hits.

**Supplementary Figure 2. Recovered snoRNA-rRNA hybrids overlapping known rRNA methylation sites.**

All non-identical snoRNA chimeras mapped to rRNA were tabulated. For each snoRNA, numbers are indicated for all interactions overlapping 2’-O-methylation site. (A) Numbers recovered in the current study. (B) Previously reported numbers of snoRNA-rRNA interactions ( Kudla *et al.*, 2011). This dataset contained 25,486 distinct hybrids, of which 3,995 are snoRNA-rRNA hybrids. Within this dataset, we found snoRNA-rRNA hybrids for 37 of the 43 C/D box snoRNAs reported to methylate yeast rRNA in the SnOPY database. For 27 of these snoRNAs, hybrids overlapped the cognate rRNA methylation site.

**Supplementary Figure 3. RDN37 chimeric hits over SNR4.**

(A) Yeast grown in synthetic medium containing 2% glucose. (B) Yeast transferred to synthetic medium containing 2% EtOH/Glycerol for 20 min.

**Supplementary Figure 4. Wild type and mutant U3 strains.**

(A) Wild type U3 (SNR17A/B) interactions over rDNA locus RDN37. (B) RDN37 interactions over SNR17A gene (intron included). (C) Growth rates of strains expressing wild-type U3, the mutant U3, or depleted for U3, following transfer to glucose medium.

**Supplementary Figure 5.**

(A) Growth curve (OD600) of snr4∆, snr45∆, HISMX6-PGAL1-SNR45 snr4∆, HISMX6-PGAL1-SNR4 snr45∆ and wild type in minimal medium containing 2% w/v glucose. A total of three biological replicates were grown, with three technical replicates analyzed for each. A key indicating strains is shown to the right of each panel. (B) snR45-18S rRNA hybrid from yeast CLASH. The acetylated cytosine is indicated in red. Watson-Crick base-pairs, denoted by a blue line; G-U base-pairings are denoted by a dot. Dashed line denotes the boundary between snoRNA and rRNA regions of the hybrid. Hybrid drawn from full chimeric sequence from NOP1 CLASH, and base-pairing predicted using the ViennaRNA package. (C) U13-18S rRNA hybrid from human Fibrillarin CLASH data. (D) Alignment between snR45 and its fungal and human homologues. Conserved C and D boxes are indicated along with the sequence identified in the hybrid with 18S rRNA flanking A1773. Red indicates conservation of ≥90%, blue indicates ≥50% conservation, and black is <50% conserved.

**Supplementary Figure 6. Interactions between snR4 and 18S rRNA.**

(A, B) snR4-18S rRNA hybrids from CLASH. The acetylated cytosine is indicated in the hybrid drawn from full chimeric sequence from Nop1p/Fibrillarin CLASH, and base-pairing predicted using the ViennaRNA package. (C) Alignment between snR4 and fungal homologues. Conserved C and D boxes are indicated along with the sequence identified in the hybrid with 18S rRNA flanking A1280. Red indicates conservation of ≥90%, blue indicates ≥50% conservation, and black is <50% conserved.

**Supplementary Figure 7. Correlations for all reproducible mRNA hybrids.**

(A) Correlation heatmap. (B) Correlation between single hits and chimeric hits. (C) Correlation between hybrid hits and mRNA expression.

**Supplementary Table 1. Oligonucleotides used in this work.**

**Supplementary Table 2. snoRNA-rRNA interactions at rRNA methylation sites.**

Column A: snoRNA - the name of the known snoRNA involved in the interaction. Column B: subunit - rRNA subunit involved in the interaction. Column C: coordinate - the position of the methylated nucleotide in the rRNA subunit. Column D: Overlapping (O/L) ( Kudla *et al.*, 2011) - number of hybrids overlapping the methylation site (obtained from data reported by Kudla *et al.*, 2011). Column E: Overlapping (O/L) this study – number of hybrids overlapping the methylation site (obtained from the current study). Column F: Kudla *et al.*, (2011), methylating - number of hybrids overlapping with the methylation site that can support methylation of the rRNA target nucleotide (obtained from Kudla *et al.*, 2011 data). Column G: this study, methylating - number of hybrids overlapping with the methylation site that can support methylation of the rRNA target nucleotide (obtained from the current study). Column H: blocking_current_meth_snoRNAs - number of chimeras formed by the “cognate” snoRNA (known to bind at this site, as in column A) but unable to guide methylation and thus blocking the correct base pairing at the site. Column I: blocking_range_meth_snoRNAs – the name of the snoRNA guiding methylation at the neighboring site and thus blocking the base pairing of the cognate snoRNA at the site. Column J: blocking_range_meth_snoRNAs_No - number of hybrids formed by the neighboring snoRNA and thus blocking the binding of the cognate snoRNA at the site. Column K: blocking_other_meth_snoRNAs – the name of the snoRNA blocking the interaction of the cognate snoRNA but NOT guiding methylation at the neighboring site (possibly binding conventionally elsewhere in the rRNA). Column L: blocking_other_meth_snoRNAs_No – number of hybrids formed at this position by the blocking snoRNA named in Column K.

**Supplementary Table 3. All snoRNA-mRNA interactions for combined NOP1 datasets**

Excel spreadsheet listing all snoRNA-mRNA interactions in the combined NOP1 datasets. The first worksheet lists all of the interactions found, along with the hybrid counts for experiments performed in glucose and ethanol in separate columns. The second and third worksheets show clusters of hybrid hits in the combined glucose and ethanol datasets, created using the combine_hyb_merge script from the hyb package ( Travis *et al*., 2014). The cluster descriptions include the gene based co-ordinates of each fragment (where each gene includes a 20 base pair flank on each end).

**Supplementary Table 4. All snoRNA-mRNA interactions for combined NOP proteins**

Excel spreadsheet listing all snoRNA-mRNA interactions in the combined NOP protein datasets. The first worksheet lists all of the interactions found, along with the hybrid counts, for all hybrids, and reproducible hybrids with a predicted binding energy below -12dG. The second worksheet shows clusters of hybrid hits in the set of reproducible hybrids. The clusters were generated using the same method as in Supplementary Table 3.

**Supplementary Table 5. snoRNA-mRNA potentially directing RNA methylation.**

Column A: methsite_id – position of the potentially methylated nucleotide in the target mRNA. Column B: hybrid_snoRNA – the name of the snoRNA potentially guiding methylation. Column C: hybrid_count – the number of snoRNA-mRNA chimeras supporting potential methylation at the site. Column D: experiment_count – the number of individual experiments from which the hybrids were obtained. Column E: box – the name of the snoRNA box upstream of which the interaction is taking place.
