# Supplementary figures and images for: Mapping targets for small nucleolar RNAs in yeast
Source: Wellcome Open Res. 2018 Nov 22;3:120. Originally published 2018 Sep 19. [Version 2] doi: 10.12688/wellcomeopenres.14735.2 (PMC6171561; doi:10.12688/wellcomeopenres.14735.2)

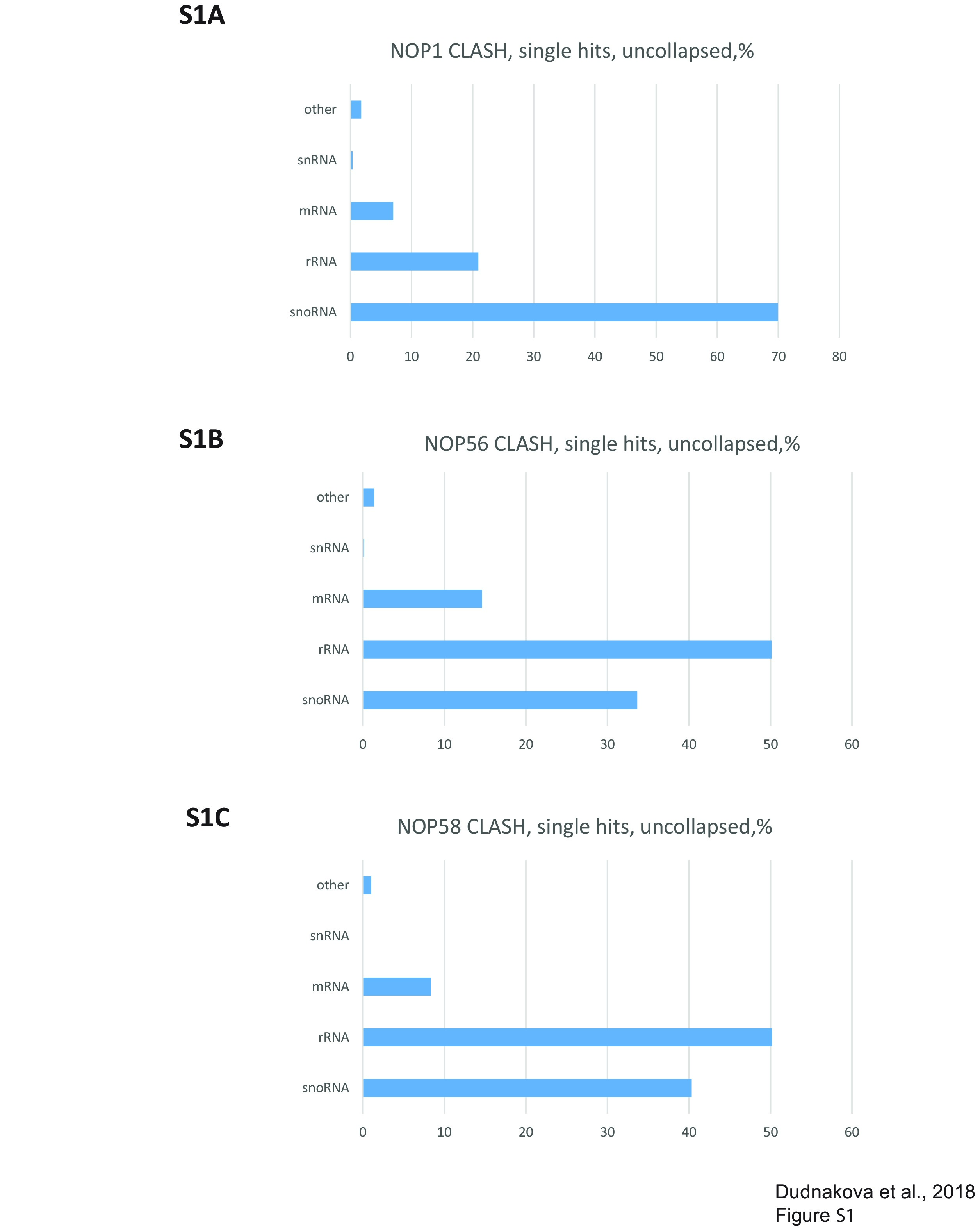

Supplement: Supplementary file 1 [file wellcomeopenres-3-16240-s0000.tgz › 258a7923-6ab6-4083-b268-e7f6e780c211_Supplementary_Figure_S1.eps.jpg]

## Recovered snoRNA-rRNA hybrids overlapping known rRNA methylation sites

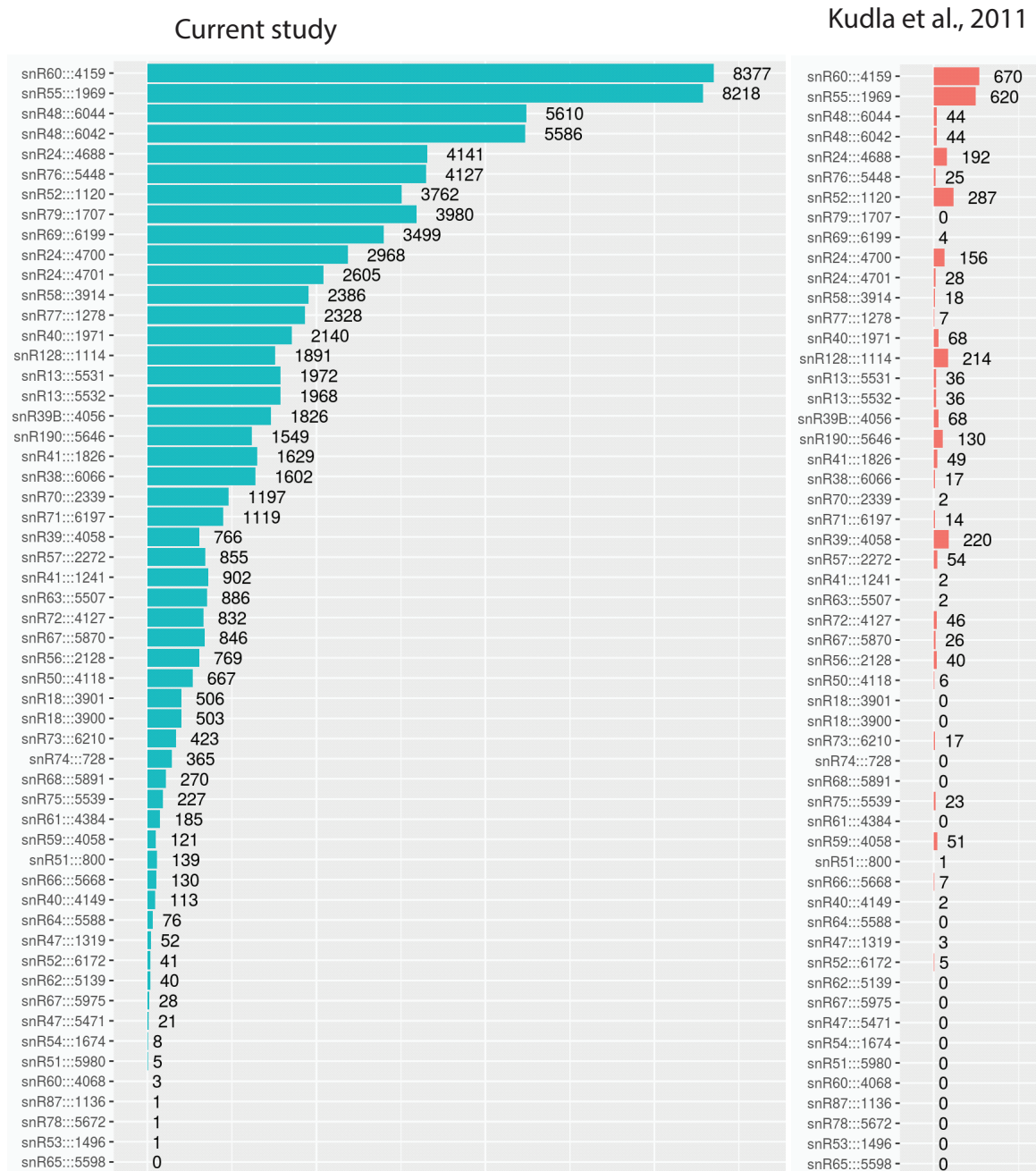

Supplement: Supplementary file 2 [file wellcomeopenres-3-16240-s0001.tgz › 2d462b07-33a3-48af-8ac8-77dd6dd31138_Supplementary_Figure_2.pdf]

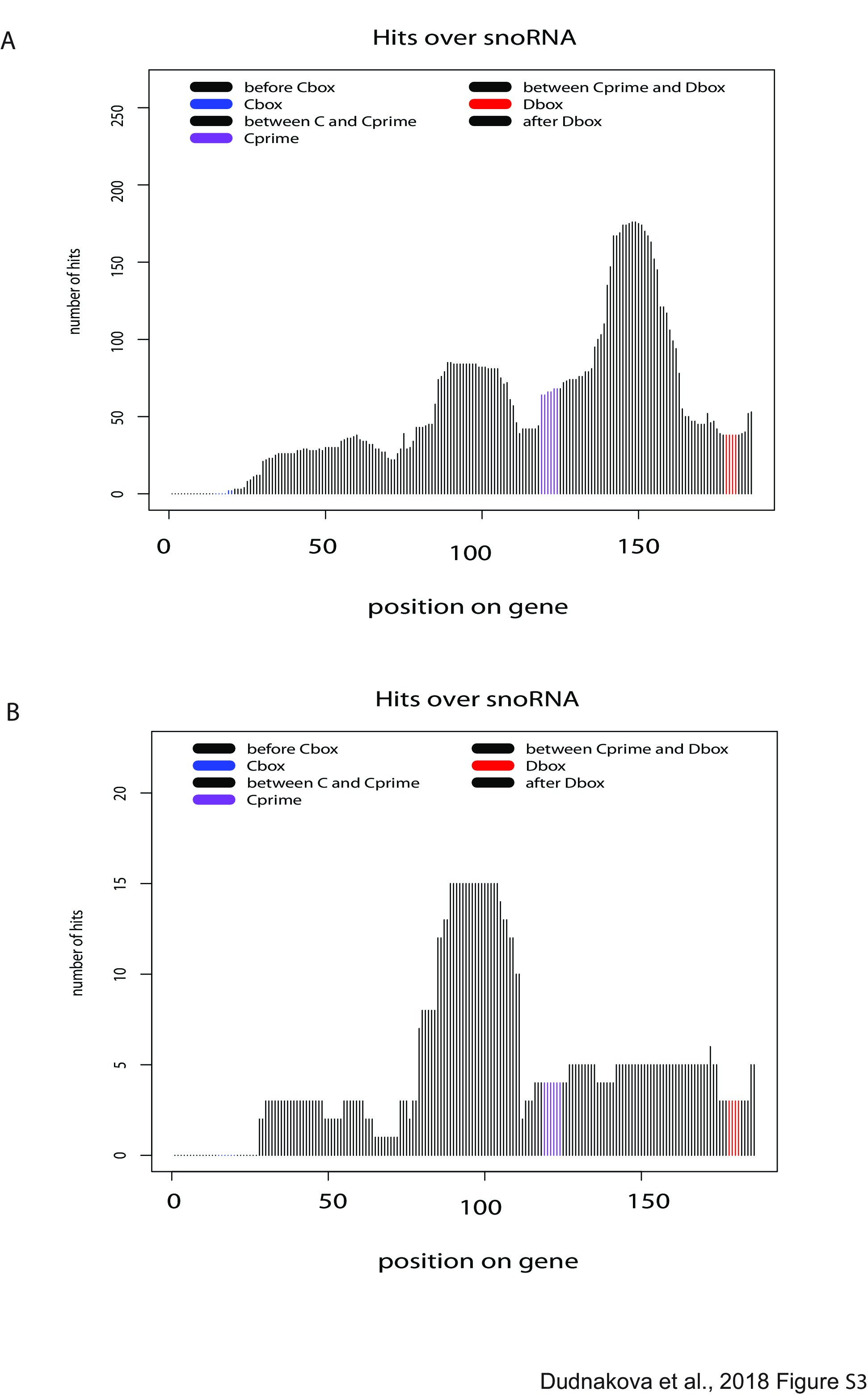

Supplement: Supplementary file 3 [file wellcomeopenres-3-16240-s0002.tgz › 6247b133-d292-41b8-8911-083abcd2b517_Supplementary_Figure_S3.eps.jpg]

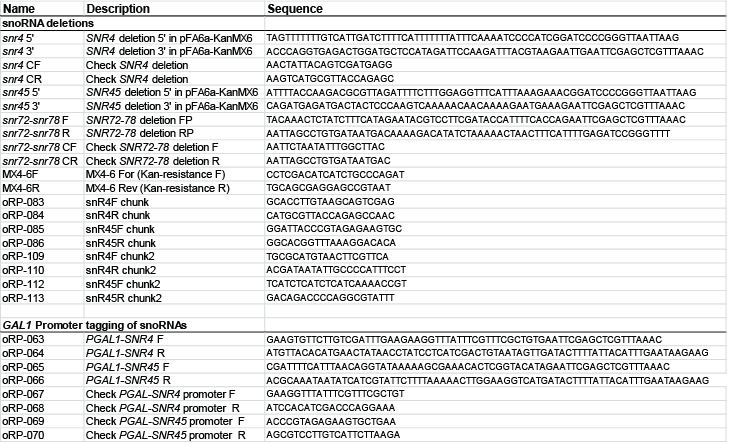

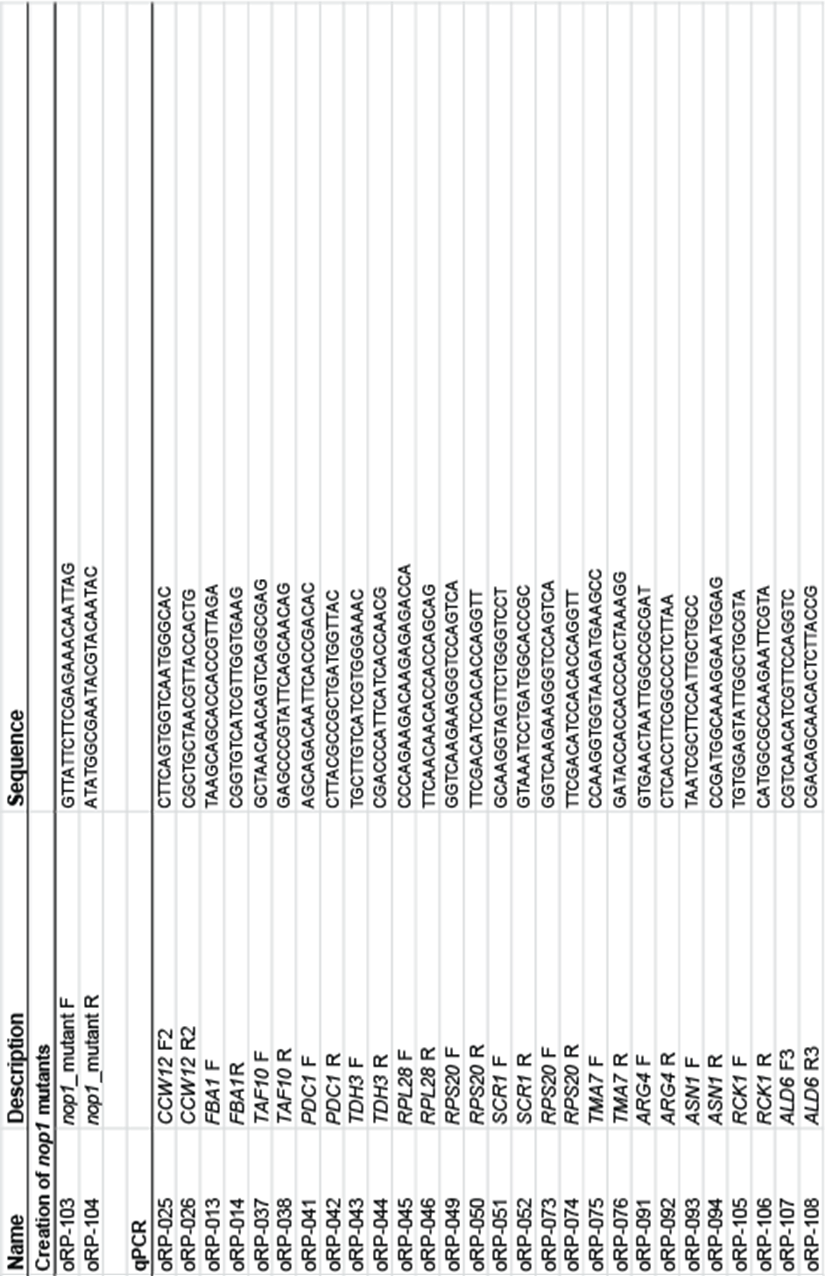

Supplement: Supplementary file 8 [file wellcomeopenres-3-16240-s0007.tgz › ee22c4fc-80b1-4761-8192-2834ff7abc0d_Supplementary_Table_1_.docx]
