# Supplementary material for: Mapping targets for small nucleolar RNAs in yeast
Source: Wellcome Open Res. 2018 Nov 22;3:120. Originally published 2018 Sep 19. [Version 2] doi: 10.12688/wellcomeopenres.14735.2 (PMC6171561; doi:10.12688/wellcomeopenres.14735.2)
Supplement: Peer review history

# Peer review: Referee response for version 2

**DOI:** 10.21956/wellcomeopenres.16240.r34323

**Published:** 2018-11-26

**Authors:** Lars Wieslander

**Affiliations:** 1 Department of Molecular Biosciences, The Wenner-Gren Institute, Stockholm University, Stockholm, Sweden

I have read this submission. I believe that I have an appropriate level of expertise to confirm that it is of an acceptable scientific standard.

# Peer review: Referee response for version 1

**DOI:** 10.21956/wellcomeopenres.16053.r33939

**Published:** 2018-10-22

**Authors:** Pascale Romby

**Affiliations:** 1 CNRS, Architecture and Reactivity of RNA (ARN), UPR 9002, University of Strasbourg, Strasbourg, France

RNA-RNA pairings are playing numerous roles in living organisms across kingdoms. Their identification, monitoring their dynamics and their functional inputs are still challenging tasks. Recent works enable at the genomic scale the mapping of RNA-RNA interactions using crosslinking followed by ligation of RNA hybrids and high-throughput sequencing. Such a method has been used in this work to map the targets of the box C/D small nucleolar RNAs using key protein partners (Nop1, Nop56 and Nop58) as the baits. This study identifies large sets of pairings involving most of the known snoRNAs and supports previous findings that the primary function of C/D box snoRNAs is to induce methylation at specific nucleotides of rRNAs. However, this work reveals unexpected findings such as the binding of snoRNAs at rRNA sites, which are not modified, or the binding between two snoRNAs. Interestingly, the CLASH approach was also conducted on the RNA helicase Mtr4 and the data revealed that minority of snoRNA-rRNA interactions predicted to guide methylation was recovered. This allows the authors to propose that Mtr4 is aimed to dissociate inappropriate snoRNA-rRNA interactions. Although the manuscript did not show any mechanistic studies validating the mechanisms of action of snoRNA on mRNAs for instance, the work is certainly of interest and will be useful for the community working on snoRNAs. It opens also new ideas and concepts of RNA regulation. 

 Minor comments:

 1- Most of the snoRNAs were recovered by the CLASH approach except snR53, snR65 and snR78. Is there an explanation? Are these three snoRNAs expressed at low levels or under peculiar conditions of growth? 

 2- The data obtained on Mtr4 are really of interest. However this is not clear to me whether the other types of interactions (snoRNA-snoRNA, snoRNA-mRNA) were also identified as possible targets for Mrt4. This might also provide some clues whether these potential interactions might be inappropriate or are endowed with regulatory functions. 

 3- More discussion on the snoRNA-mRNA interactions would be appropriate. Do specific snoRNA target mRNAs that are functionally related? It is claimed that some of the mRNAs might be modified. Do these modifications take place in the coding regions or in the UTRs of the mRNA? The authors have mentioned that most snoRNA-mRNA duplexes are predicted to represent structural interactions. They should clarify what do they mean by structural interactions. 

 4- The deletion of snR4 or mutation in U3 caused alteration of the steady state yields only of a few number of mRNA targets. Deletion of snR4 decreases the levels of target mRNAs while U3 mutations causes both enhanced and decreased levels of mRNA targets. Is there a possible correlation between the position of the snoRNA binding site on the mRNA and their effect on the mRNA yield?

I have read this submission. I believe that I have an appropriate level of expertise to confirm that it is of an acceptable scientific standard.

# Peer review: Referee response for version 1

**DOI:** 10.21956/wellcomeopenres.16053.r33942

**Published:** 2018-10-08

**Authors:** Arlen W. Johnson, Joshua Black

**Affiliations:** 1 University of Texas at Austin, Austin, TX, USA; 2 Department of Molecular Biosciences, University of Texas at Austin, Austin, TX, USA

In this paper, the authors present a large amount of CLASH data and bioinformatics analysis on RNA hybrids that crosslink to Nop1, Nop56, Nop58 and Mtr4. The sequencing depth allows them to identify numerous unexpected RNA duplexes formed by various snoRNAs. The data could be a resource for others interested in novel functions of snoRNAs but as is, the paper is largely descriptive. Several comments regarding the work:

**Major points:**

1. There has been some speculation that snoRNA directed modification of mRNAs under stress conditions results from off targeting when ribosome production is reduced. Does the data presented reveal anything to address this idea?

 

1. Related to Figure 4. Panel A – nucleotides should all be ribonucleotides if this is RNA. The Ts should be Us in the expanded image on the right. Was there any rationale for inserting snR77 sequence into U3? Does the U3mut enter into the SSU Processome? Its phenotype appears to mimic that of cells lacking U3. This leads us to question whether the U3mut enters the SSU Processome. I did not find results to support the statement “We demonstrate that U3 binding in 18S is functionally important.” However, this is well established in previously published work from this lab.
2. Related to Figure 5. The authors propose a model that Mtr4 has a role in unwinding non-cognate snoRNA-rRNA duplexes. This has also been proposed for Prp43. Some comparison with Prp43 would be instructive.
3. The conclusion that Mtr4 is removing snoRNAs comes from comparing the Mtr4 CLASH hybrids are compared to Nop1 hybrids. However, a better comparison would be Nop1 hybrids +/- Mtr4 or Mtr4 hybrids +/-Rrp6 or other exosome mutants. In addition, Nop1 gives 100- to 1000-fold higher reads for hybrids than does Mtr4. What is to be made of the very low recovery of hybrids in the Mtr4 sample?

 

1. The sections beginning Depletion of U3 and Depletion of snR4 are redundant with earlier sections and should be rearranged to avoid this.

 

**Minor comments:**

1. In the last paragraph of page 10, the authors state, “Strains carrying *snr4Δ* or *snr45Δ* showed no detectable growth defect relative to the isogenic wild-type (Supplementary Figure 4D and Dudnakova et al., 2018).” There is no Supplementary Figure 4D.
2. Also, the last line of page 10: “It seemed possible that snR4 and snR45 might have redundant functions, and we therefore generated double mutant strains carrying *HISMX6-PGAL1::SNR45 snr4Δ *and *HISMX6-PGAL1- SNR4 **snr45***.”

This should read “ *snr45∆*”.

We have read this submission. We believe that we have an appropriate level of expertise to confirm that it is of an acceptable scientific standard.

# Peer review: Referee response for version 1

**DOI:** 10.21956/wellcomeopenres.16053.r33938

**Published:** 2018-10-03

**Authors:** Carla Columbano Oliveira

**Affiliations:** 1 Department of Biochemistry, Institute of Chemistry, University of Sao Paulo, São Paulo, Brazil

In this article, the authors show the use of a crosslinking and sequencing of hybrids (CLASH) approach to analyze snoRNA-rRNA interactions and determine the targets of heretofore considered orphan snoRNAs. In addition, the authors identified snoRNAs binding sites to rRNAs that may compete with cognate snoRNA binding, thereby inhibiting rRNA methylation.

 To identify box C/D snoRNP targets, the authors used tagged C/D core proteins Nop1, Nop56 and Nop58 under control of their endogenous promoters. The results show that most of the targets corresponded to rRNA methylation sites, as expected. Interestingly, the same kind of experiments performed with tagged Mtr4 showed non-cognate rRNA regions, raising the hypothesis that this RNA helicase could be responsible for displacing non-cognate snoRNAs.

 The snoRNA-snoRNA interactions identified here are suggested to represent regulatory regions in pre-snoRNAs, which could be important for retention, or compartmentalization of snoRNAs.

 The authors also report snoRNA-mRNA interactions, including some that might promote methylation of the target mRNAs. These results are very interesting and could assign function to as yet considered orphan snoRNAs.

 In summary, this article shows the results of large-scale assessment of box C/D snoRNPs interactions, revealing known and additional targets of these snoRNPs, which can suggest a much broader role for box C/D complexes in posttranscriptional control of gene expression.

 Suggestions and some points for discussion:

 Introduction, pg. 3:

 “Each region is bound by a copy of Nop1, so the regions flanking either box D, box D’ or both can function as methylation guides. The strict requirement for a long region of prefect complementarity that extends to box D/D’ for guide function implies that strong snoRNA base pairing could occur without eliciting target RNA methylation.”

 Which region? Sentence should be modified for clarity.

 Typo: perfect

 Fig. 2A:

 In the case of competing snoRNAs of box C/D binding to rRNA, it would be interesting to check whether there are resulting alternative methylation sites on rRNAs.

 Fig. 4:

 The roles of U3 snoRNP and Rrp9 in pre-rRNA have already been described by the Tollervey group and others. Therefore, the relevance of the results shown in Fig 4 were not entirely clear to me. I suggest clarification of this part.

 Interestingly, however, Nop1 seems to bind more efficiently to U3m than to WT U3, although the authors considered it to bind both snoRNAs with equal affinity. The effect is even more evident when considering the fact that Rrp9 does not bind U3 mut.

 Page 10, snR4 and snR45:

 Was the hypothesis that these two snoRNAs could have redundant functions based only on the observation that they are not essential? This should be better explained.

 Pg 17: “We predict that Mtr4 plays an important surveillance role in dissociating incorrect but stable snoRNA interactions.”

 The hypothesis of Mtr4 displacing non-cognate snoRNA-rRNA binding is very interesting, but it is not clear to me how Mtr4 would distinguish between correct and incorrect stable interactions.

 Fig. 5D:

 Color coding is confusing.

 Fig. 6:

 Other than the pre-snoRNAs snR17A and 17B that undergo splicing, have the snoRNAs isolated here been shown to be transcribed as longer precursor RNAs that are processed to shorter mature RNAs? From the figure, I gather that some of them are longer at the 5’ region, while others are longer at the 3’ region.

 snoRNA-mRNA interactions: is there any evidence that mRNAs are methylated by Nop1? If mRNAs are methylated by Nop1, this modification should take place in the nucleolus, where Nop1 is localized. Would the authors predict that mRNAs are directed to the nucleolus for methylation?

 What would be the mechanism by which snR4 controls levels of mRNAs?

I have read this submission. I believe that I have an appropriate level of expertise to confirm that it is of an acceptable scientific standard.

# Peer review: Referee response for version 1

**DOI:** 10.21956/wellcomeopenres.16053.r33941

**Published:** 2018-10-03

**Authors:** Lars Wieslander

**Affiliations:** 1 Department of Molecular Biosciences, The Wenner-Gren Institute, Stockholm University, Stockholm, Sweden

I think that this article reports sound results. The authors explore a method previously introduced (partly) by the authors to comprehensively map interactions between snoRNAs and various other RNAs, especially rRNA. These data considerably add to our current knowledge about snoRNA function. I have very little to say about the data. Materials and methods are extensive, possibly somewhat difficult to follow in relation to the results. For example, quantitative PCR comes up twice as does RNA library preparation. Perhaps some help to relate to what parts of the results could be given. Also, in some parts the descriptions are detailed (for example polyA selection), while other parts could give some more information (for example, I would have liked to know the hybridization temperature and washing conditions for Northerns). A very small correction: “in functionally” to “is functionally” on page 10, right column, 6 line from end of next to last paragraph.

I have read this submission. I believe that I have an appropriate level of expertise to confirm that it is of an acceptable scientific standard.

# Peer review: Referee response for version 1

**DOI:** 10.21956/wellcomeopenres.16053.r33940

**Published:** 2018-10-01

**Authors:** Bruno Charpentier, Mathieu Rederstorff

**Affiliations:** 1 CNRS (French National Center for Scientific Research), Molecular Engineering and Articular Physiopathology (IMoPA), University of Lorraine, Nancy, France

The research article by Dudnakova *et al.* from the D. Tollervey lab reports the observation of RNA targets for snoRNAs in yeast, using the CLASH sequencing technique. The issue addressed is of great importance to understand the activity of ribonucleoproteins carrying non coding RNAs that serve as a guide for these activities. The article is well written and scientifically sound.

 Most of the reported data are confirmative of previous knowledge in *S. cerevisiae*, bringing only limited novel information, but which however proves the robustness of the approach to isolate snoRNA-RNA targets hybrids. Indeed, among the ≈190000 reproducible hybrids retrieved, a large majority corresponded to snoRNAs interactions with rRNAs, as expected, covering most of the known methylation sites in rRNAs. Very interestingly also, two recently described acetylation sites for the snoRNAs snr4 and 45 were detected by the method.

 Nevertheless, what constitutes the most exciting part of the article is that the authors also observed possible novel RNA targets for snoRNAs, as well as putatively novel functions. Some hybrids revealed a possible competitive regulatory role for snoRNAs, when they were bound to non-cognate targets. Additionally, the observation of snoRNA/snoRNA hybrids suggests possible cross-regulation between snoRNAs. Interestingly, several hybrids also corresponded to snoRNA/mRNA pairs.

 Finally, when CLASH was performed using the helicase Mtr4 rather than C/D box snoRNA core proteins, the snoRNA-rRNA hybrids retrieved corresponded to fewer bona-fide methylation sites, suggesting a possible role for Mtr4 in dissociating non-cognate snoRNA/rRNA interactions.

 A large amount of work was accomplished, but the article is rather descriptive and hypothetical. Very interesting aspects are evocated but on which we can only conjecture, such as regulation functions for snoRNA/snoRNA snoRNA/mRNA hybrids or snoRNA/non cognate rRNA hybrids, or the hybrids obtained with Mtr4. No experimental data are yet provided to confirm these assumptions.

Clarifications and minor points:

- The authors mention that some hybrids correspond to non-cognate snoRNAs bound to rRNA methylation sites, possibly blocking methylation. Could the authors clarify this interesting aspect? How does the snoRNAs bind these non-cognate targets?
- The reasons why the authors inserted the box D flanking region of snR77 in the U3 mutant, nor the consequences of this mutation when yeast cells were grown on Glc medium, are not clear. An equivalent mutant was described by Samarsky & Fournier ^1^ carrying truncation of the three helices (U3del). What is the advantage of substitution with snR77 sequence rather than helix 3 deletion? What is the mRNA target of snR77? Could the authors comment on Rrp9 binding and U3 function/yeast viability taking into account their data and preceding data on *in vitro* and *in vivo* Rrp9 binding on U3 and U3 mutants ^2^^-^^4^.
- snoRNA-snoRNA hybrids revealed potential interactions between mature snoRNAs and precursor forms of target snoRNAs. Figure 6 shows a preferred location in the 3’ sequence of the targeted precursor form. The authors could comment on one exception, snr128, which is produced as a bicistronic precursor with snR190.
- pp3, beginning of the second paragraph: it could be interesting to give for non-specialist readers WEB sites dedicated to snoRNA databases.
- pp10, paragraph 2: the authors mention 12% of snoRNA-snoRNA hybrids and then refer to 0.3%. Can they comment on that or does the 0.3% value rather refers to snRNAs?
- Construction of the U3 mutant with conditional expression is partially described at two places in Results section (in page 10 and page 17). It would be good to correct this redundancy and to provide clarification. For instance, it is not clear for non-specialists why as described in page 10, the mutant strain is inviable in Glc medium: the existence of the second SNR17B gene is not clearly explained.
- pp10, last paragraph, the first sentence refers to Supplementary Figure 4D which apparently does not exist.
- It would be good to make homogeneous the writing of the name of the U3 mutant. i.e. U3mut VS U3 mut VS U3m.
- Figures and supp. Figures are of poor resolution.

 

Typographic errors:

- pp3, first paragraph, lane 22: prefect should be replaced by perfect.
- pp3, at different places in the Methods section a space must be added between the values and the units.
- pp13, first sentence of the second paragraph was probably truncated (Comparison of SNR4 …).
- pp17, second paragraph on the right: BPS should be replaced by PBS.

We have read this submission. We believe that we have an appropriate level of expertise to confirm that it is of an acceptable scientific standard.

## References

[1] SamarskyDAFournierMJ: Functional mapping of the U3 small nucleolar RNA from the yeast Saccharomyces cerevisiae. *Mol Cell Biol*.1998;18(6) :3431-44 958418310.1128/mcb.18.6.3431PMC108924

[2] RothéBManivalXRollandNCharronCSenty-SégaultVBranlantCCharpentierB: Implication of the box C/D snoRNP assembly factor Rsa1p in U3 snoRNP assembly. *Nucleic Acids Res*.2017;45(12) : 10.1093/nar/gkx424 7455-7473 10.1093/nar/gkx424 28505348PMC5499572

[3] CléryASenty-SégaultVLeclercFRauéHABranlantC: Analysis of sequence and structural features that identify the B/C motif of U3 small nucleolar RNA as the recognition site for the Snu13p-Rrp9p protein pair. *Mol Cell Biol*.2007;27(4) : 10.1128/MCB.01287-06 1191-206 10.1128/MCB.01287-06 17145781PMC1800722

[4] GrannemanSPruijnGJHorstmanWvan VenrooijWJLuhrmannRWatkinsNJ: The hU3-55K protein requires 15.5K binding to the box B/C motif as well as flanking RNA elements for its association with the U3 small nucleolar RNA in Vitro. *J Biol Chem*.2002;277(50) : 10.1074/jbc.M206631200 48490-500 10.1074/jbc.M206631200 12381732
